# Pan-Cancer Analyses Reveal Genomic Features of FOXM1 Overexpression in Cancer

**DOI:** 10.3390/cancers11020251

**Published:** 2019-02-21

**Authors:** Carter J Barger, Connor Branick, Linda Chee, Adam R. Karpf

**Affiliations:** 1 Eppley Institute for Cancer Research, University of Nebraska Medical Center, Omaha, NE 68198, USA; carter.barger@ucsf.edu (C.J.B.); connor.branick@unmc.edu (C.B.); Linda.Chee@unmc.edu (L.C.); 2 Fred & Pamela Buffett Cancer Center, University of Nebraska Medical Center, Omaha, NE 68198, USA

**Keywords:** FOXM1, pan-cancer, gene amplification, genomic instability, Retinoblastoma protein Cyclin E1, fallopian tube epithelial cells, testicular germ cell tumors, high-grade serous ovarian cancer, basal breast cancer

## Abstract

FOXM1 is frequently overexpressed in cancer, but this has not been studied in a comprehensive manner. We utilized genotype-tissue expression (GTEx) normal and The Cancer Genome Atlas (TCGA) tumor data to define FOXM1 expression, including its isoforms, and to determine the genetic alterations that promote FOXM1 expression in cancer. Additionally, we used human fallopian tube epithelial (FTE) cells to dissect the role of Retinoblastoma (Rb)-E2F and Cyclin E1 in FOXM1 regulation, and a novel human embryonic kidney cell (HEK293T) CRISPR FOXM1 knockout model to define isoform-specific transcriptional programs. FOXM1 expression, at the mRNA and protein level, was significantly elevated in tumors with FOXM1 amplification, p53 inactivation, and Rb-E2F deregulation. *FOXM1* expression was remarkably high in testicular germ cell tumors (TGCT), high-grade serous ovarian cancer (HGSC), and basal breast cancer (BBC). FOXM1 expression in cancer was associated with genomic instability, as measured using aneuploidy signatures. FTE models confirmed a role for Rb-E2F signaling in FOXM1 regulation and in particular identified Cyclin E1 as a novel inducer of FOXM1 expression. Among the three *FOXM1* isoforms, *FOXM1c* showed the highest expression in normal and tumor tissues and cancer cell lines. The CRISPR knockout model demonstrated that FOXM1b and FOXM1c are transcriptionally active, while FOXM1a is not. Finally, we were unable to confirm the existence of a FOXM1 auto-regulatory loop. This study provides significant and novel information regarding the frequency, causes, and consequences of elevated FOXM1 expression in human cancer.

## 1. Introduction

FOXM1 is a member of the Forkhead box (FOX) transcription factor family, which is unified by a conserved winged helix DNA binding motif [1]. FOXM1 plays a key role in proliferation and cell cycle progression through transcriptional activation of a G2/M-specific gene network [2,3,4]. Increased FOXM1 gene expression and its transcriptional signature are detected in many cancer types [5,6,7,8,9]. Notably, pan-cancer analysis identified FOXM1 as the top gene associated with poor prognosis [10]. FOXM1 is also a promising therapeutic target for cancer treatment [11,12,13,14,15]. While increased FOXM1 expression is reported in many cancers, we lack an understanding of how FOXM1 copy number, mRNA, and protein expression compare and are associated in pan-cancer. 

Human *FOXM1* has ten exons, with alternative splicing of exons Va and VIIa, giving rise to three variants: FOXM1a, FOXM1c, and FOXM1b. FOXM1b and -c are transcriptional activators, while FOXM1a was reported to be transcriptionally inactive [16]. While other cancer-specific transcripts have been reported for FOXM1 [17,18], only FOXM1a, -b and -c have been consistently investigated for their expression and function [19,20,21,22,23,24,25,26]. Previous studies of *FOXM1* isoform expression in cancer have reported that either *FOXM1b* or *FOXM1c* has the highest level of expression; however, these studies use different methods and have small sample sizes. Additionally, studies of *FOXM1* isoform expression have focused on a single or limited number of cancer types, warranting a need for a comprehensive analysis using normal and cancer tissues. In this regard, the first comprehensive comparison of *FOXM1* isoform expression employed genotype-tissue expression (GTEx) normal, The Cancer Genome Atlas (TCGA) cancer, and Target Cancer datasets [27], and validated the findings of others showing that *FOXM1c* has the highest expression followed by *FOXM1b* and *FOXM1a* [25,26,28]. However, it is still uncertain if FOXM1 isoforms show a differential increase in expression between paired normal and tumor samples. Furthermore, FOXM1 isoforms have been assigned various functions and transcriptional activities but these studies were limited to luciferase promoter assays or targeted gene expression studies [16,21,29,30]. 

Herein, we: 1) investigated changes in and links between FOXM1 copy number, mRNA, and protein expression across TCGA cancers; 2) compared *FOXM1* expression in cancer vs. normal tissues; 3) identified key genomic features that contribute to increased FOXM1 expression using both pan-cancer analyses and engineered cell models; and 4) determined the association between FOXM1 expression and cancer genomic instability. Furthermore, we determined *FOXM1* isoform expression in GTEx and TCGA normal and TCGA cancer tissues, TCGA paired normal and tumor samples, and human immortalized and cancer cell lines. Finally, we characterized the transcriptional activity of the three major FOXM1 isoforms in a newly developed FOXM1 knockout cell line, using luciferase assays and RNA-sequencing. Together, these data provide significant new understanding of the nature of FOXM1 deregulation in cancer, including its causes and potential consequences. The findings presented are also relevant for targeting FOXM1 in cancer, both by revealing vulnerable cancer types and by identifying potential biomarkers for therapeutic studies.

## 2. Results

### 2.1. FOXM1 Expression in Pan-Cancer 

**Figure 1:**
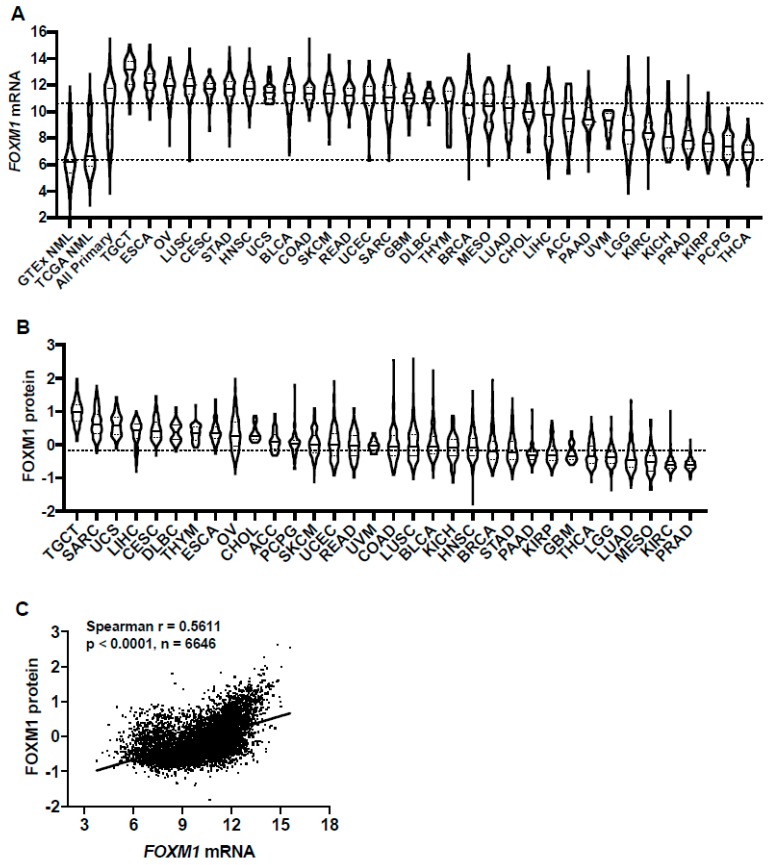
FOXM1 mRNA and protein expression in genotype-tissue expression (GTEx) normal, The Cancer Genome Atlas (TCGA) normal, and TCGA cancer tissues. (**A**) *FOXM1* mRNA expression (RNA-seq RSEM, log2(norm count +1)) in GTEx and TCGA datasets. Sample lines represent medians and quartiles. Low and high dotted lines across the graph represent median expression for GTEx and TCGA normal, and TCGA cancer, respectively. Cancer types consist of primary tumors only and are ranked by median *FOXM1* mRNA expression. The key to all TCGA abbreviations is shown in Table S1. (**B**) FOXM1 protein expression (Reverse Phase Protein Array; RPPA, pan-can normalized) across TCGA cancer types. (**C**) *FOXM1* mRNA expression (RNA Seq V2 RSEM, log2(norm count +1) correlations with FOXM1 protein expression (RPPA, pan-cancer normalized)) across TCGA cancer types.

FOXM1 is over-expressed in many human cancers [31]. Previous studies of FOXM1 expression in cancer have used different methods, are limited to small sample sizes, and/or have focused on a single or limited number of cancer types. To provide a more comprehensive evaluation of FOXM1 expression in cancer, we compared *FOXM1* mRNA expression across 32 TCGA cancer types (Table S1) and in TCGA and GTEx normal tissues, using UCSC TOIL to correct for batch effects and to allow for sample merging [27]. *FOXM1* mRNA expression was increased in all TCGA cancer types compared to GTEx and TCGA normal (Figure 1A). Comparing across cancer types, there was a broad spectrum of *FOXM1* expression, suggesting that high *FOXM1*-expressing cancers may have unique genetic features that drive increased *FOXM1* expression (Figure 1A). Furthermore, based on the interquartile range, the spread of *FOXM1* expression varied in some cancer types more than others; e.g., breast cancer (BRCA) has a wide spread while testicular germ cell tumors (TCGT) have a narrow spread (Figure 1A), which could be due to some cancers consisting of more than one clearly defined subtype and therefore having more genetic diversity. We also compared FOXM1 protein expression across the same TCGA cohorts. Similar to *FOXM1* mRNA, FOXM1 protein expression varied across tumor types and showed a broad spectrum of expression (Figure 1B). Importantly, FOXM1 mRNA and protein expression were highly correlated in pan-cancer (Figure 1C), suggesting an important role for gene expression regulation in determining FOXM1 protein expression and its functional status in cancer. 

### 2.2. FOXM1 Overexpression in Pan-Cancer Is Associated with FOXM1 Amplification and Copy Number Gain

**Figure 2:**
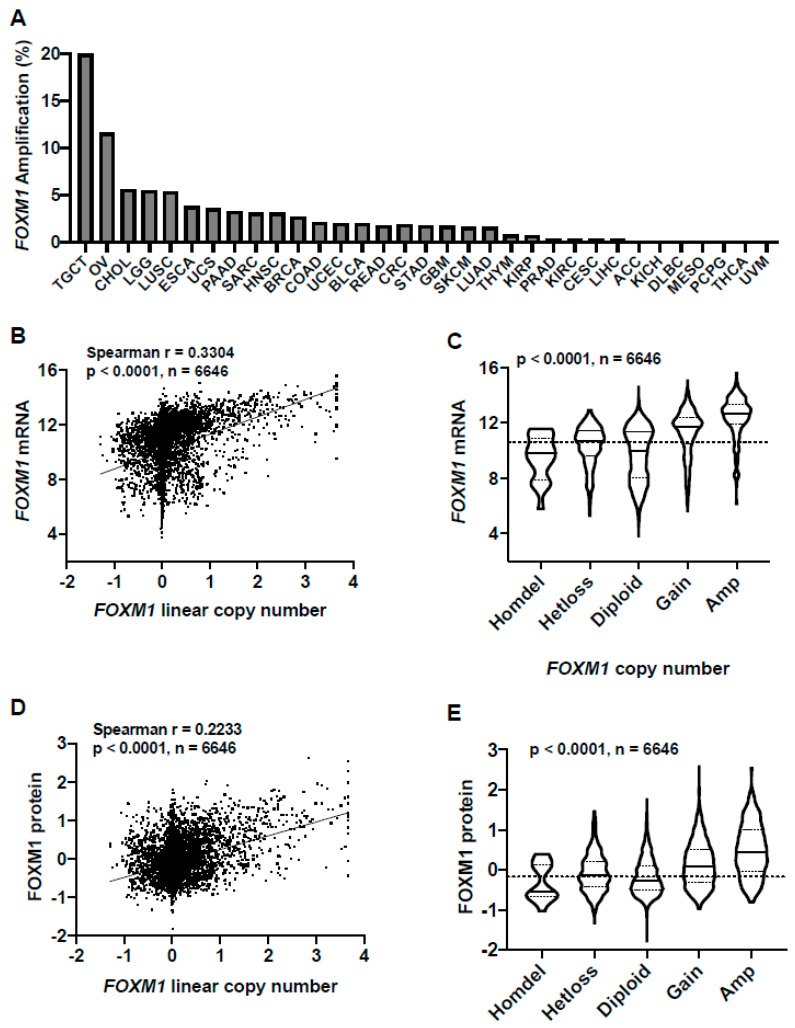
*FOXM1* amplifications in TCGA pan-cancer and correlations with FOXM1 mRNA and protein expression. (**A**) *FOXM1* amplification frequency in TCGA cancer types as determined by GISTIC. Cancer types are ranked by *FOXM1* amplification frequency. (**B**) *FOXM1* mRNA expression (RNA-seq RSEM, log2(norm count +1)) compared to *FOXM1* linear copy number values across all TCGA cancer types. (**C**) *FOXM1* mRNA expression (RNA-seq RSEM, log2(norm count +1)) compared to *FOXM1* copy number (GISTIC) across all TCGA cancer types. The *p* value for analysis of variance (ANOVA) with post-test for linear trend is shown. Sample lines represent medians and quartiles. The dotted line across the graph represents the median expression for all TCGA primary tumors. (**D**) FOXM1 protein expression (RPPA) correlated with *FOXM1* linear copy number values across all TCGA cancer types. (**E**) FOXM1 protein expression (RPPA) compared to *FOXM1* copy number across all TCGA cancer types. The *p* value for ANOVA with post-test for linear trend is shown. Sample lines represent medians and quartiles. The dotted line across the graph represents median expression for all TCGA primary tumors.

To understand the mechanisms driving increased FOXM1 expression in TCGA pan-cancer, we first focused on *FOXM1* copy number alterations. *FOXM1* is located at chromosome 12p13.33, an amplified region in cancer [25,32,33,34]. We previously reported that *FOXM1* amplification correlates with *FOXM1* mRNA expression in high-grade serous ovarian cancer (HGSC) [25]. We determined the frequency of *FOXM1* amplification across the 32 TCGA cancer types using the TCGA Genomic Identification of Significant Targets in Cancer (GISTIC) copy number dataset (Figure 2A). When considering *FOXM1* GISTIC-defined amplifications plus copy number gains, a significantly higher proportion of tumors from each type were affected (Figure S1). To determine if *FOXM1* copy number correlates with FOXM1 expression, we compared *FOXM1* copy number with FOXM1 mRNA and protein expression in TCGA primary tumors. We observed a progressive increase in both FOXM1 mRNA and protein expression with increased copy number, which was highly significant (Figure 2B–E).

We previously reported that *FOXM1* copy number is associated with increased FOXM1 expression in HGSC cell lines [25]. To extend this comparison to cancer cell lines more generally, we used Cancer Cell Line Encyclopedia (CCLE) data [35]. Similar to primary tumors, FOXM1 mRNA and protein expression showed a highly significant correlation in the CCLE (Figure S2A). Moreover, just as in primary tumors, there was a progressive increase in both FOXM1 mRNA and protein expression with copy number, which was highly significant (Figure S2B–E). Table S2 shows compiled FOXM1-omics data for CCLE lines, which provides a useful resource for investigators wishing to select appropriate experimental models for study.

Notably, amongst all tumor types with TCGA data, *FOXM1* was most frequently amplified in testicular germ cell tumors (TGCT), which is also the cancer type showing the highest level of FOXM1 mRNA and protein expression (Figure 2A; Figure 1A,B). We also noted that some cancer types that lack *FOXM1* amplifications have lower FOXM1 expression (e.g., THCA, PCPG) (Figure 2A; Figure 1A,B). In contrast, other cancer types lacked *FOXM1* amplification but had FOXM1 expression at or above the pan-cancer median (e.g., CESC, DLBC) (Figure 2A; Figure 1A,B), indicating that additional genetic alterations may contribute to increased FOXM1 expression in cancer. 

### 2.3. FOXM1 Overexpression in Pan-Cancer Correlates with Genomic Instability 

**Figure 3:**
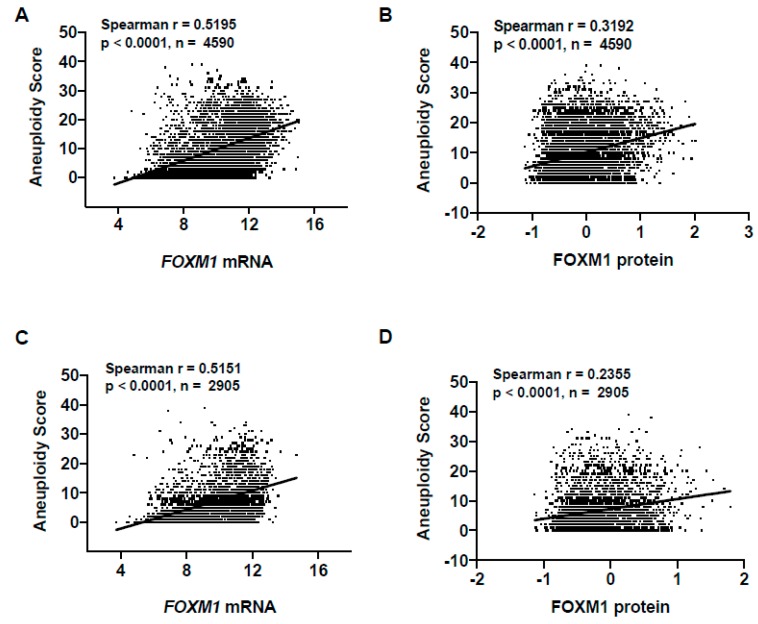
Association of FOXM1 expression with aneuploidy in TCGA pan-cancer. (**A**) *FOXM1* mRNA expression (RNA-seq RSEM, log2(norm count +1)) correlations with tumor aneuploidy scores in TCGA primary tumors. (**B**) FOXM1 protein expression (RPPA, pan-can normalized) correlation with tumor aneuploidy score in TCGA primary tumors. (**C**) *FOXM1* mRNA expression (RNA-seq RSEM, log2(norm count +1)) correlation with tumor aneuploidy score in *FOXM1* diploid TCGA primary tumors. (**D**) FOXM1 protein expression (RPPA, pan-can normalized) correlations with tumor aneuploidy scores in *FOXM1* diploid TCGA primary tumors.

*FOXM1* mRNA expression and the FOXM1 transcriptional program have been reported to be enriched in tumors with genomic instability [36,37,38]. To test the relationship between FOXM1 expression and genomic instability using TCGA pan-cancer data, we correlated FOXM1 expression with genomic instability using a recently defined tumor aneuploidy score [39]. In this context, aneuploidy is defined as somatic copy number alterations of whole chromosomes and chromosome arms. Thus, the aneuploidy score reflects the total number of chromosome arms with arm-level copy-number alterations in a sample. Consistent with previous work, we observed a strong association between FOXM1 mRNA and protein expression with genomic instability (Figure 3A,B). To control for *FOXM1* copy number increases in aneuploid tumors as a confounding variable, we performed the same analysis only using *FOXM1* diploid samples. Importantly, we again observed a strong association between FOXM1 expression and genomic instability (Figure 3C,D). Next, we compared FOXM1 expression using previously reported TCGA pan-cancer aneuploidy clusters [38]. Surprisingly, group 10, which is characterized by moderate aneuploidy, showed the highest *FOXM1* expression (Figure S3A). This aneuploidy cluster is enriched for TGCT, which shows *FOXM1* amplification or copy number gains in ~100% of tumors (Figure S1). *FOXM1* is located at 12p13.33; 12p gains are pathognomonic for TGCT [40], and isochromosome 12p gains occur in 87% of TGCT [41], likely accounting for increased FOXM1 expression in this cancer type. As expected, aneuploidy groups 1, 2, 3 and 5, which show high aneuploidy, were enriched for cancer types with the highest *FOXM1* mRNA expression, while group 7, which has low aneuploidy, was enriched for cancer types that show low *FOXM1* mRNA expression (Figure S3A,B; Figure 1A). The assignment of *FOXM1*-linked aneuploidy clusters to *FOXM1* diploid tumors resulted in an almost identical pattern of cluster enrichment [42]. Finally, FOXM1 protein expression correlated with a highly similar pattern of aneuploidy clusters and tumor types as seen for *FOXM1* mRNA (Figure S3C,D).

### 2.4. FOXM1 Overexpression in Pan-Cancer Correlates with TP53 and RB1 Alterations

**Figure 4:**
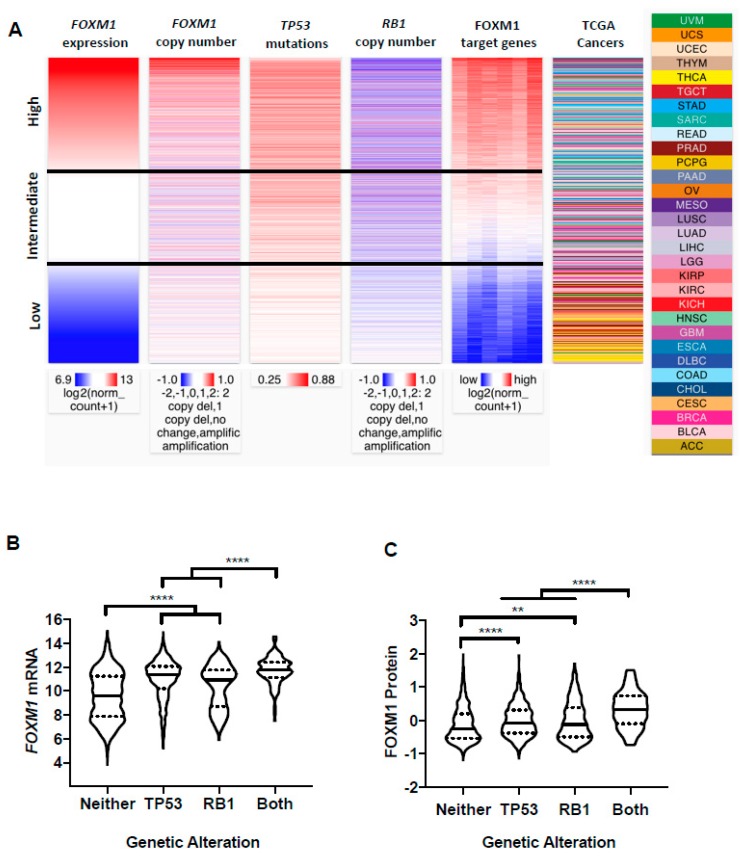
FOXM1 expression in TCGA cancer types by p53/Rb status. (**A**) Comparison of *FOXM1* mRNA expression, *FOXM1* copy number, *TP53* somatic mutations, *RB1* copy number, and FOXM1 target genes among all TCGA primary tumors with overlapping genomics data. Data were retrieved from UCSC Xena. FOXM1 target genes included *AURKB*, *CCNA2*, *CCNB1*, *CCNB2* and *CENPA*. (**B**,**C**) FOXM1 expression in tumors with *T53* somatic mutations and *RB1* copy number loss. (**B**) *FOXM1* mRNA expression (RNA-seq RSEM, log2(norm count +1)) in tumors with *T53* somatic mutations (*n* = 1288), *RB1* copy number loss or somatic mutation (*n* = 190), both alterations (*n* = 128) or neither alteration (*n* = 3362). (**C**) FOXM1 protein expression (RPPA pan-can normalized) in tumors with *T53* somatic mutations (*n* = 1288), *RB1* copy number loss or somatic mutation (*n* = 190), both alterations (*n* = 128), or neither alteration (*n* = 3362). Mann-Whitney test *p* values are shown. *p* value designations: **** < 0.0001, ** < 0.01,

Recently, *TP53* mutations, Rb-E2F deregulation, and *FOXM1* amplification or expression were found to be enriched in high aneuploidy breast tumors [43]. Prior in vitro studies have also linked *TP53* and *RB1* loss to increased FOXM1 expression [25,44,45,46]. Therefore, we used UCSC Xena Browser Heat Maps to visualize the relationship between *FOXM1* mRNA expression, *FOXM1* copy number, *TP53* mutations, *RB1* copy number, and FOXM1 target gene expression across all TCGA primary tumors. Notably, TCGA primary tumors with high *FOXM1* expression demonstrated more frequent *TP53* mutations, *RB1* copy number loss, and activation of FOXM1 target genes (Figure 4A). Additionally, tumors with intermediate *FOXM1* expression but *FOXM1* diploid or copy number loss tended to have *TP53* mutations and *RB1* loss, while tumors with low *FOXM1* expression displayed *FOXM1* diploid or copy number loss, wild-type *TP53*, and diploid *RB1* (Figure 4A). To formally test these associations, we grouped TCGA primary tumors by *TP53* and *RB1* status and compared FOXM1 mRNA and protein expression levels. We observed the highest FOXM1 mRNA and protein expression in tumors with alterations in both *TP53* and *RB1*, with either single alteration giving an intermediate phenotype (Figure 4B,C). These data implicate p53 and Rb-E2F deregulation as key drivers of FOXM1 overexpression in pan-cancer. 

### 2.5. High-Grade Serous Ovarian Cancer (HGSC) and Basal Breast Cancer (BBC) Have Highly Similar FOXM1 Alterations

**Figure 5:**
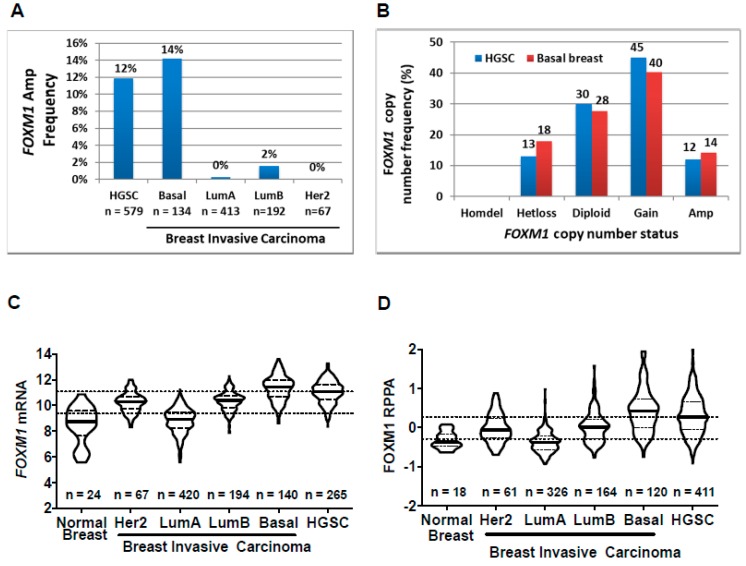
*FOXM1* copy number alterations (CNA) and expression in TCGA OV (HGSC) vs. BRCA (Breast) molecular (PAM50) subtypes. (**A**) *FOXM1* amplification frequency in HGSC vs. breast molecular subtypes. (**B**) *FOXM1* copy number status in HGSC vs. basal breast. (**C,D**) FOXM1 expression in HGSC vs breast molecular subtypes (**C**) *mRNA* expression (RNA-seq) and (**D**) protein expression (RPPA, pan-can normalized). Lines represent group medians. Low and high dotted lines across the graph represent median expression for Her2, LumA and LumB breast molecular subtypes, and HGSC, respectively.

TCGA reported that HGSC and BBC show similar genomic features, including ubiquitous *TP53* mutations and increased genomic instability in the form of copy number alterations, as compared to other subtypes of breast cancer and other cancer types [6,47]. We hypothesized that basal breast cancer (BBC) may show similar FOXM1 alterations to HGSC. However, in TCGA pan-cancer data, breast cancer (BRCA) showed a lower frequency of FOXM1 mRNA, protein expression, and genomic amplifications compared to HGSC (Figure 1A,B; Figure 2A). Because TCGA BRCA data do not differentiate between breast cancer subtypes, we retrieved TCGA PAM50 expression subtype data for TCGA breast cancer and categorized samples based on molecular subtype. As hypothesized, BBC and HGSC showed similarly increased frequency of *FOXM1* amplifications compared to other molecular subtypes of breast cancer (Figure 5A). In addition, there was a striking similarity in *FOXM1* copy number alteration frequency between BBC and HGSC (Figure 5B). BBC and HGSC showed consistently increased FOXM1 mRNA and protein expression compared to other breast cancer molecular subtypes and normal breast tissue (Figure 5C,D). These data reveal a similar spectrum of FOXM1 alterations in BBC and HGSC, further underscoring the similar molecular phenotype of these tumors [6,47].

### 2.6. Deregulation of the Rb-E2F Pathway, Including Cyclin E1 Expression, Promotes FOXM1 Overexpression in Fallopian Tube Epithelial (FTE) Cells, An HGSC Precursor Cell Model

**Figure 6:**
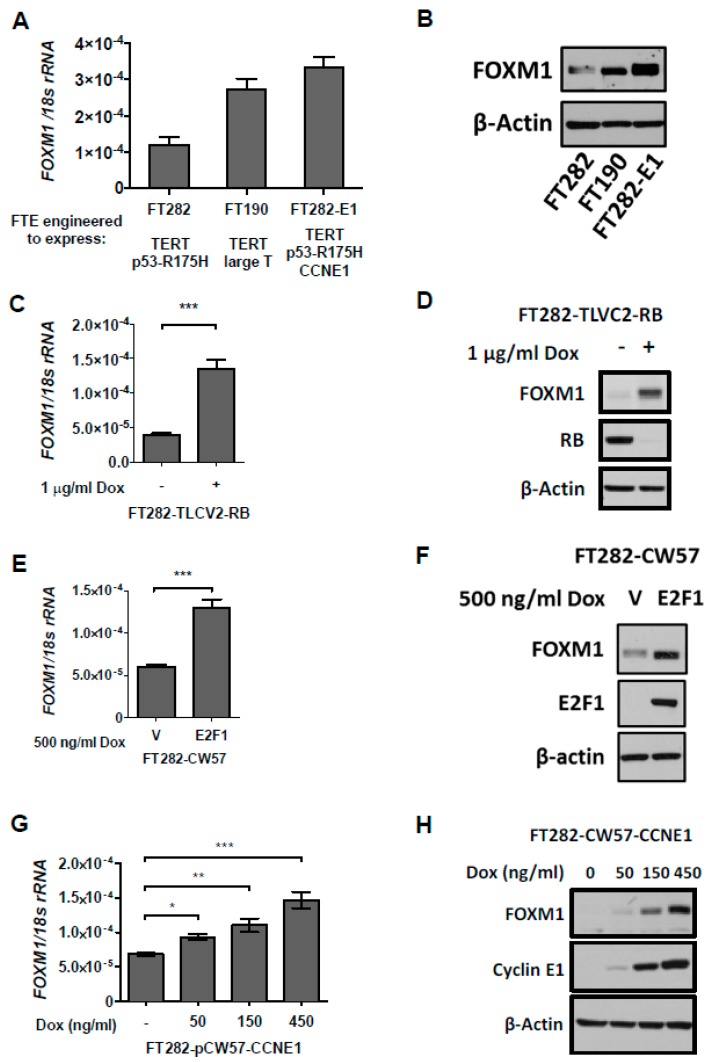
FOXM1 expression in fallopian tube epithelial (FTE) cells engineered for deregulation of the Rb-E2F pathway. (**A**,**B**) FOXM1 mRNA and protein expression were measured by RT-qPCR and Western blot, respectively, in the indicated FTE cell lines. β-actin is shown as a loading control. (**C**,**D**) FOXM1 expression by RT-qPCR and Western blot, respectively, in RB1 CRISPR KO FT282 cells. (**E**,**F**) FOXM1 expression by RT-qPCR and Western blot, respectively, in E2F1 inducible FTE cells. (**G**,**H**) FOXM1 expression by RT-qPCR and Western blot, respectively, in CCNE1 inducible FTE cells. Bars represent mean ± SD. Student’s *t* test *p* values are shown. *p* value designations: *** < 0.001, ** < 0.01, * < 0.05.

We show that FOXM1 expression increases with *TP53* and *RB1* genetic alterations in pan-cancer (Figure 4). We previously reported that FOXM1 expression increases in p53 and Rb disrupted ovarian surface epithelial (OSE) cells, and observed that *FOXM1* expression decreases following E2F1 knockdown [25]. To validate these findings in a more relevant genetic context and precursor cell model for HGSC, we utilized hTERT-immortalized fallopian tube epithelial (FTE) cells [48,49]. We used FTE cells engineered to overexpress mutant p53-R175H (FT282), large T antigen (TAg) (FT190), or mutant p53-R175H + Cyclin E1 (FT282-E1). Rb-E2F deregulation via either TAg or Cyclin E1 expression in FTE cells increased FOXM1 mRNA and protein expression compared to FTE cells expressing mutant p53 alone (Figure 6A,B). Notably, Cyclin E1 expressing cells showed the highest expression of FOXM1, revealing Cyclin E1 as a novel upstream regulator of FOXM1. Next, we introduced temporal genetic modifications to the Rb-E2F pathway into FT282 cells. We modified the lenti-CRISPR vector into an inducible system and engineered FTE cells for *RB1* knockout using a guide RNA to human *RB1* [50,51]. We doxycycline (dox) treated the resulting cells for 72 h, then sorted cells based on positive green fluorescent protein (GFP) expression to enrich for *RB1* knockout. We expanded the cells without dox for one week prior to harvesting. Importantly, we observed a significant increase in FOXM1 mRNA and protein expression with RB knockout (Figure 6C,D). In addition, we observed increased expression of both FOXM1 mRNA and protein when E2F1 was inducibly overexpressed (Figure 6E,F). 

**Figure 7:**
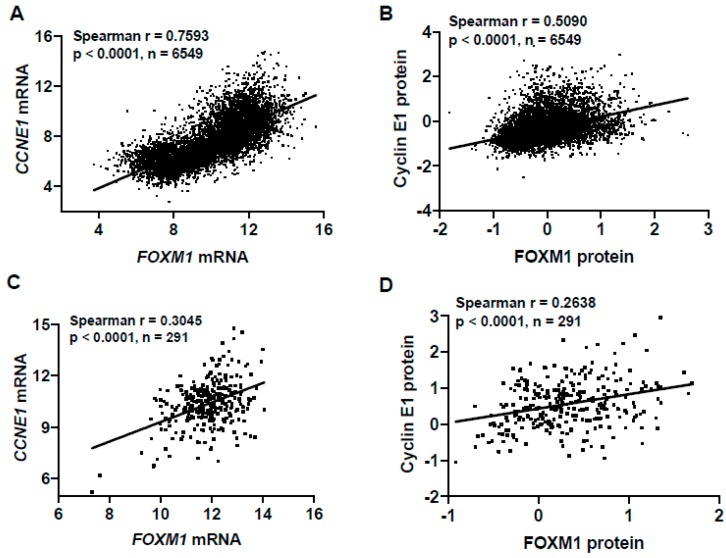
FOXM1 and CCNE1 expression correlation in pan-cancer and high-grade serous ovarian cancer (HGSC) tissues. (**A**) Correlation of *FOXM1* expression and *CCNE1* mRNA expression (RNA-seq RSEM, log2(norm count +1)) in TCGA pan-cancer tissues. (**B**) Correlation of FOXM1 expression and CCNE1 protein expression ((RPPA, pan-can normalized) in TCGA pan-cancer tissues. (**C**) Correlation of *FOXM1* expression and *CCNE1* mRNA expression (RNA-seq RSEM, log2(norm count +1)) in TCGA HGSC tissues. (**D**) Correlation of FOXM1 expression and CCNE1 protein expression (RPPA, pan-can normalized) in TCGA HGSC tissues.

*CCNE1* is a frequently amplified oncogene in HGSC, resulting in its overexpression [52]. To confirm our observation of increased FOXM1 expression in FTE cells with constitutive Cyclin E1 expression, we overexpressed Cyclin E1 in FTE cells in a dox-inducible manner, and observed a dose-dependent increase in FOXM1 (Figure 6G,H). Conversely, FOXM1 expression in FTE cells did not result in increased Cyclin E1 [42]. Additionally, we observed a highly significant correlation between FOXM1 and *CCNE1* mRNA and Cyclin E1 protein expressions in TCGA pan-cancer and HGSC primary tumor data (Figure 7). These data provide mechanistic support to our observation that FOXM1 expression is increased in tumors with defects in p53 and Rb/E2F pathways. In addition, these studies reveal that Cyclin E1 is an upstream activator of FOXM1 expression in human cancers, including HGSC.

### 2.7. FOXM1 Isoform Expression in GTEx Normal, TCGA Normal, and TCGA Pan-Cancer Tissues 

**Figure 8:**
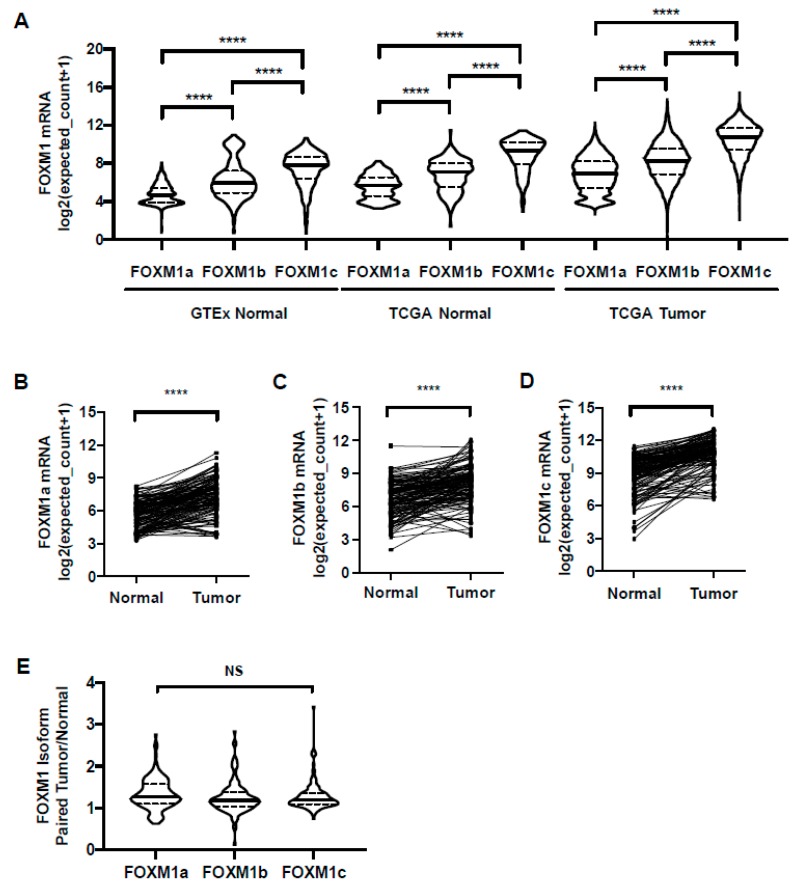
*FOXM1* isoform expression in GTEx normal, TCGA normal, and TCGA cancer tissues. (**A**) *FOXM1* isoform expression (RNA-seq RSEM, log2(norm count +1)) in GTEx (*n* = 843) and TCGA normal (*n* = 175) and cancer tissues (*n* = 6309). (**B**–**E**) *FOXM1* isoform expression (RNA-seq RSEM, log2(norm count +1)) in TCGA paired normal and tumor tissues (*n* = 135). (**B**) *FOXM1a* mRNA expression (RNA-seq RSEM, log2(norm count +1)). (**C**) *FOXM1b* mRNA expression (RNA-seq RSEM, log2(norm count +1). (**D**) *FOXM1c* mRNA expression (RNA-seq RSEM, log2(norm count +1)). (**E**) *FOXM1* isoform mRNA expression (RNA-seq RSEM, log2(norm count+1)) ratio between paired tumor and normal tissue (*n* = 135).

FOXM1 has three known splice variants: FOXM1a, b, and c, which encode proteins with varying activities [16,53,54]. Current knowledge of *FOXM1* isoform expression in different cancer types is inconsistent and has not been defined in relation to normal tissues or paired normal and tumor samples. To address these questions, we determined *FOXM1* isoform expression in normal and tumor tissues using TOIL GTEx and TCGA RNA-seq datasets from UCSC Xena. We found that *FOXM1c* was the predominant isoform expressed in both normal and tumor tissues, followed by *FOXM1b* and *FOXM1a* (Figure 8A). Additionally, all three *FOXM1* isoforms had increased expression in paired tumor vs. normal samples (Figure 8B–D). Comparing the ratios of isoform expression between tumor and normal, all three isoforms showed a similar increase in expression in tumors (Figure 8E). Thus, the observed increase in overall FOXM1 expression in cancer is not due to differential expression of any particular isoform. We next used the CCLE cancer cell line RNA-seq dataset to compare *FOXM1* isoform expression, and again found that *FOXM1c* showed the highest expression (Figure S4A). Finally, using RT-qPCR with isoform-specific primers, we observed that *FOXM1c* was the predominantly expressed isoform in a panel of cancer cell lines, followed by *FOXM1b* and *FOXM1a* (Figure S4B) [25]. Together, these data provide strong evidence that *FOXM1c* is the highest expressed *FOXM1* isoform in both normal tissues and cancer.

### 2.8. FOXM1 Isoforms b and c Have Similar Transcriptional Activity

**Figure 9:**
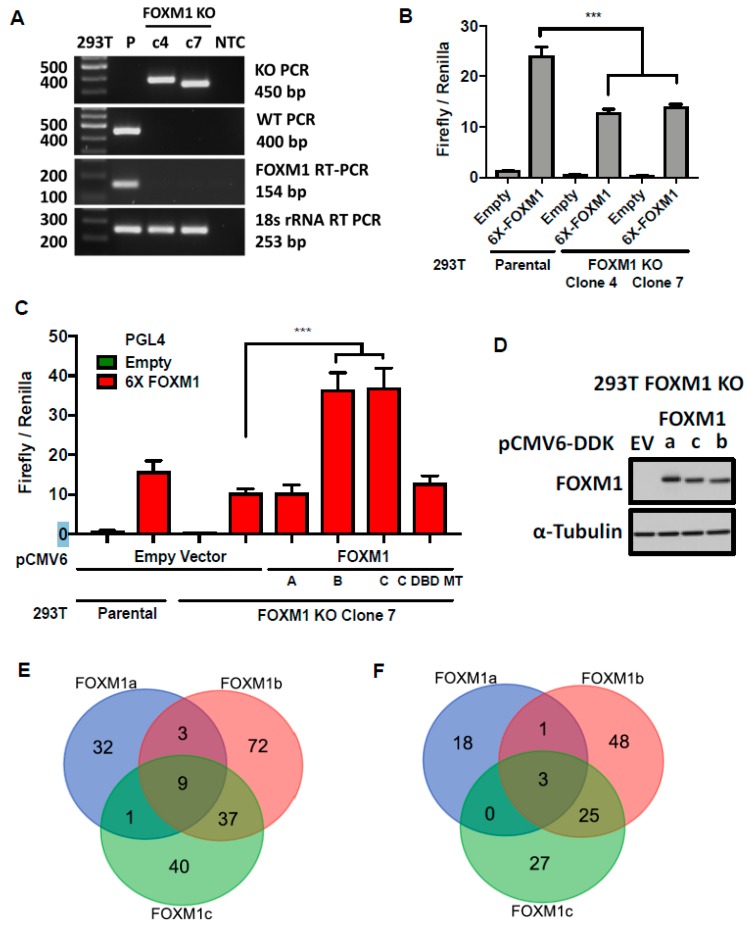
FOXM1 isoform transcriptional activity in HEK293T (293T) FOXM1 knockout cells. (**A**) PCR genotype of two *FOXM1* CRISPR knockout clones. (**B**) 6X-FOXM1 reporter assay in 293T *FOXM1* knockout clones. (**C**–**F**) 293T *FOXM1* knockout clone 7 transiently reconstituted with FOXM1 isoforms a, b and c. (**C**) 6X-FOXM1 reporter assay. (**D**) FOXM1 Western blot related to RNA-seq. (**E**–**F**) Venn diagrams of gene transcripts showing significant changes in expression for each FOXM1 isoform. (**E**) Overlap of gene transcripts showing those with both significant increases and decreases (FDR < 0.05) in expression. (**F**) Overlap of transcriptional targets showing only those with significant (FDR < 0.05) increases in expression. Bars represent mean ± SD. Student’s *t* test *p* values are shown. *p* value designations: *** < 0.001.

FOXM1 encodes several transcripts, two of which (b and c) are the predominant isoforms used in most experimental studies. To understand how increased FOXM1 isoform expression in cancer relates to function, we used CRISPR to knock out *FOXM1* in 293T cells (Figure 9A). We validated two FOXM1 knockout cell clones using the 6X-FOXM1 luciferase reporter assay, and observed the knockout clones showed significantly decreased reporter activity compared to parental cells (Figure 9B). We next tested the ability of FOXM1 isoforms to rescue FOXM1 reporter activity and observed that FOXM1 isoforms b and c were capable of rescue, but FOXM1a and a FOXM1c DNA binding domain mutant (DBD MT) were not (Figure 9C). We additionally confirmed the transcriptional activity of FOXM1 isoforms in FT282 cells, which have low endogenous FOXM1 expression (Figure 6), and observed that FOXM1b and FOXM1c activated the 6X-FOXM1 reporter, while FOXM1a and a FOXM1c DBD MT did not (Figure S5). To extend these observations, we reconstituted a 293T FOXM1 knockout clone with FOXM1a, b, or c (Figure 9D) and performed RNA-seq (Table S3). We compared transcriptional changes, including both significant increases and decreases (False Discovery Rate (FDR) < 0.05), associated with FOXM1 isoform reconstitution and observed that FOXM1c and b produced the greatest overlap in transcriptional targets (Figure 9E; Table S4). When comparing only genes that showed a significant increase in expression (FDR < 0.05), we again observed greatest overlap between FOXM1c and FOXM1b, and minimal overlap with FOXM1a (Figure 9F; Table S5). Collectively, these data confirm that FOXM1c and FOXM1b are transcriptionally active, but that FOXM1a is either inactive or shows substantially less activity. 

### 2.9. Lack of Evidence for A FOXM1 Auto-Regulatory Loop

Finally, we examined a previously reported FOXM1 positive auto-regulatory loop [55,56], initially by using 293T FOXM1 knockout cells and a FOXM1 promoter reporter assay. Unexpectedly, we did not observe a significant difference in FOXM1 promoter activity when comparing 293T parental and FOXM1 KO cells (Figure S6A). Consistent with this, Kuramochi HGSC cells with dox-inducible FOXM1c expression showed *CCNB1* activation (a canonical FOXM1 target gene), but not increased endogenous *FOXM1* expression (Figure S6B,C). Moreover, dox-inducible expression of either FOXM1b or –c activated *CCNB1* but not endogenous *FOXM1* expression in both hOSE and FT282 cells (Figure S6D). Conversely, overexpression of the FOXM1c DBD MT, which functions as a dominant negative, resulted in a significant decrease in *CCNB1* mRNA expression, but no change in *FOXM1* endogenous mRNA expression in FT282 cells (Figure S6D). Finally, transient overexpression experiments in 293T cells showed that FOXM1 b or -c induced *CCNB1* but not endogenous *FOXM1*, while FOXM1a lacked transcriptional activity (Figure S6E,F). Together, these data do not support the existence of a FOXM1 auto-regulatory loop, at least in the cell types examined.

## 3. Discussion

While FOXM1 over-expression is reported in many different cancers, there has not been a comprehensive pan-cancer analysis of FOXM1 status. To address this, we performed a pan-cancer analysis of FOXM1 across 32 TCGA cancer types compared to TCGA normal and GTEx normal tissues. Importantly, in TCGA, increased *FOXM1* mRNA expression corresponded to both increased FOXM1 protein expression and increased target gene expression, showing that FOXM1 is functionally active in these tumors. Another notable finding was that *FOXM1* copy number alterations contribute to FOXM1 overexpression. For example, TGCT (i.e., testicular germ cell tumors) showed the highest FOXM1 expression level and *FOXM1* amplification frequency among TCGA cancer types. Our analyses revealed cancer types with distinct levels of *FOXM1* copy number alterations and expression, including HGSC and BBC, which have highly elevated FOXM1, and THCA and PCPG, which show remarkably lower FOXM1. Notably, within BRCA, only BBC showed increased FOXM1 expression, emphasizing the importance of genomics in classifying tumors for therapeutic targeting. Consistent with this, a recent study identified FOXM1 as a valuable therapeutic target in BBC [57], a disease, like HGSC, that is in need of new therapeutic strategies. The data presented here provide useful knowledge for prioritizing the study of FOXM1 function and therapeutic targeting in different types of human cancer. 

Pan-cancer analyses demonstrated that FOXM1 expression is increased in TCGA tumors with p53 mutations and Rb loss of function, and we validated these findings using genetic modeling of p53, Rb/E2F1, and Cyclin E1 in FTE cells. In particular, we identified Cyclin E1 as an upstream activator of FOXM1 expression. In cell models, Cyclin E1 induced FOXM1 but the reverse was not true, and TCGA data sets showed significant associations between Cyclin E1 and FOXM1 expression. Cyclin E1/CDK2 complexes inactivate Rb by phosphorylation, leading to E2F activation [58], which may mechanistically explain why Cyclin E1 induces FOXM1 expression, but this requires testing. Moreover, TP53 mutations are an early or initiating event in HGSC, and Cyclin E1 and FOXM1 expression are detected in HGSC precursor lesions [52,59,60]. Our data thus suggest that FOXM1 expression may contribute to early oncogenic events in HGSC development and progression, alone or in combination with Cyclin E1, potentially including the induction of genomic instability. In support of this idea, forced overexpression of FOXM1 contributes to genomic instability in cell line models [43,61]. Pan-cancer analyses performed here also demonstrated a significant association between both FOXM1 mRNA and protein expression and aneuploidy. Importantly, these associations were retained in tumors with diploid FOXM1, suggesting that increased FOXM1 expression promotes genomic instability, rather than just representing a by-product of it.

FOXM1 has three predominant splice variants: FOXM1a, b, and c, which encode proteins with varying activities [1]. Considering the differential expression and functional potential of different FOXM1 isoforms, it is important to determine which variants are responsible for oncogenic activity. We performed a comprehensive analysis of *FOXM1* isoform expression in GTEx and TCGA normal, TCGA pan-cancer, and CCLE cell lines to determine the FOXM1 isoform expression profiles. We found that the predominant *FOXM1* isoform expressed in all contexts was FOXM1c. However, analysis of TCGA paired normal and tumor showed that all three *FOXM1* isoforms have relatively equivalent increases in paired cancer vs. normal tissues. We further investigated FOXM1 isoform transcriptional activity using cell models, FOXM1 reporter assays, endogenous target gene expression, and RNA-seq analyses. Our data confirmed that FOXM1c and FOXM1b are transcriptionally active but that FOXM1a is not, or has highly reduced activity. FOXM1 is known to interact with other proteins and to dimerize, therefore FOXM1 may also have important functions independent of its transcriptional activity. This is particularly true for FOXM1a, which is transcriptionally inactive. In addition, FOXM1 isoforms may complement each other as heterodimers to perform certain functions and possibly regulate each other through direct interaction, considering that FOXM1 is capable of dimerizing [62,63]. Finally, although we observed significant transcriptional activity for both FOXM1b and -c in a variety of experimental contexts, we could not validate the existence of a FOXM1 auto-regulatory loop, consistent with a recent report [64].

## 4. Materials and Methods

### 4.1. The Cancer Genome Atlas (TCGA), Cancer Cell Line Encyclopedia (CCLE), Genotype-Tissue Expression (GTEx) Data Sets

*FOXM1* somatic copy-number alterations were compared in provisional datasets for TCGA primary tumors obtained from cBioPortal [65]. TOIL GTEx (cell lines removed) and TCGA (normal and primary tumor only) total gene and transcript RNA-seq datasets were obtained from UCSC Xena (https://xena.ucsc.edu/) and were used for pan-normal and cancer analysis of *FOXM1* mRNA expression and *FOXM1* mRNA isoform expression (Ensembl Genome Reference Consortium release GRCh38: FOXM1a, Ensembl transcript ENST00000342628.6; FOXM1c, Ensembl transcript ENST00000359843.7; FOXM1b, Ensembl transcript ENST00000361953.7). TOIL reprocesses raw GTEx and TCGA RNA-sequencing data to correct for batch effects and to allow for the merging of samples across GTEx and TCGA datasets for pan-analyses [27]. The level 4 TCGA RPPA dataset was obtained from The Cancer Proteome Atlas [66,67] and was used for pan-cancer analysis of FOXM1 protein expression. The RPPA level 4 dataset was a reprocessing of raw TCGA RPPA data using a novel approach, called ‘replicates-based normalization’ (RBN) to correct for batch effects and ultimately allow for the merging of samples across 19 different cancer types for pan-cancer analyses [68]. TCGA aneuploidy scores and arm call files were obtained from the Genomic Data Commons Pan-Cancer Atlas [38,39]. Aneuploidy Cluster Membership was obtained from the published supplemental section [38]. The CCLE transcript RNA-seq dataset was used for comparison of *FOXM1* isoform mRNA expression in human cancer cell lines [69]. 

### 4.2. Reverse Transcriptase Quantitative PCR (RT-qPCR)

Total RNA was purified using TRIzol (Invitrogen, Carlsbad, CA, USA and RNA was DNase-treated using the DNA-free kit (Thermo Fisher Scientific, Waltham, MA, USA) or Direct-zol RNA Purification Kit (Zymo Research, Irvine, CA, USA) with in column DNase treatment. RNA quality was determined by a denaturing gel. RNA was converted to cDNA using the iScript cDNA synthesis kit (BioRad, Hercules, CA, USA). One µL of 1:5 cDNA sample dilutions were used for qPCR reactions. Standard curves were prepared using gel-purified end-point RT-PCR products. All samples were run in triplicate, and all gene expression data were normalized to 18s rRNA. PCR was performed with an annealing temperature of 60 °C and a total of 45 cycles for all primer pairs. Dissociation curves were performed to confirm specific product amplification. RT-qPCR standards for each gene were generated from a mixture of human cell cDNA via end point RT-PCR then gel purification, using the appropriate primer pair. Gradient PCR reactions were used to optimize annealing temperatures for each primer set. Primer sequences are listed in Table S6 [25]. 

### 4.3. Western Blot Analysis

Whole cell protein extracts were prepared with RIPA buffer [1× PBS, 1% NP40, 0.5% sodium deoxycholate, 0.1% sodium dodecyl sulfate (SDS)] supplemented with protease and phosphatase inhibitors (Sigma-Aldrich, St. Louis, MO, USA), and centrifuged at 4 °C for 10 min at 14,000 *g*. Protein concentration was determined by the BCA protein assay (Thermo Fisher Scientific, Waltham, MA, USA). Equal amounts of protein (30–50 µg) were fractionated on 4–12% gradient SDS-polyacrylamide gel electrophoresis gels (Invitrogen) and transferred to PVDF membrane (Roche, Basel, Switzerland). Membranes were stained with Ponceau S to confirm efficient transfer and equal loading, then blocked with 5% nonfat dry milk in Tris-buffered saline Tween-20 (TBST) for 1 h at room temperature. The membranes were incubated with primary antibodies in 5% nonfat dry milk or bovine serum albumin (BSA) in TBST at 4 °C overnight followed by incubation with secondary antibody in 5% nonfat dry milk in TBST for 1 h at room temperature. The following primary antibodies were purchased from the following suppliers and used at the indicated dilutions: Santa Cruz Biotechnology (Dallas, TX, USA)-Cyclin E1 (sc-247; 1:1000), FOXM1 (sc-271746; 1:500), E2F1 (sc-251; 1:500), β-Actin (sc-47778; 1:5000); Cell Signaling Technology (Danvers, MA, USA))-FOXM1 (#5436, 1:1000); and BD Biosciences (San Jose, CA, USA)-RB1 (#554136; 1:300). Enhanced chemiluminescence (Thermo Fisher Scientific) was used for protein detection. 

### 4.4. Cell Culture 

HGSC precursor cells or immortalized fallopian tube epithelial (FTE; FT190, FT282-E1 and FT282) cells were a generous gift from Professor Drapkin (University of Pennsylvania, Philadelphia, PA, USA) and were cultured in DMEM-Ham’s F12 50/50 (Corning Life Sciences, Tewksbury, MA, USA), supplemented with 2% USG (Pall Corporation, (New York, NY, USA) or 10% FBS and 1% pen-strep (Thermo Fisher Scientific. FT282 clone 11 (C11) cells were cultured in DMEM-Ham’s F12 50/50 (Corning) supplemented with 10% FBS and 1% pen-strep. HEK293T cells (American Type Tissue Culture Collection) were cultured in DMEM with 10% FBS and 1% pen-strep. Cell lines were authenticated by short tandem repeat (STR) analysis at the DNA Services Facility, University of Illinois at Chicago, and confirmed to be mycoplasma free by end-point PCR at the Epigenetics Core Facility, University of Nebraska Medical Center. Doxycycline inducible cells were treated every 48 h with doxycycline (Sigma, solubilized in water) unless otherwise noted. All cell lines were maintained at 37 °C in a humidified incubator with 5% CO_2_. Cell culture medium was changed every 3–5 days depending on cell density. For routine passage, cells were split at a ratio of 1:3–10 when they reached 85% to 90% confluence. 

### 4.5. Lentivirus Production and Cell Line Transduction

Replication-deficient lentivirus was produced by transient transfection of 6.0 μg psPAX2 (Addgene #12260), 2.0 μg pMD2.G (Addgene #12259), and 8.0 μg transfer plasmid into HEK293T cells in a 10 cm dish with Lipofectamine 2000 reagent (Life Technologies, Carlsbad, CA, USA), according to the manufacturer’s instructions. Viral supernatants were collected at 48 h and passed through a 0.2 μm filter. Functional titration was performed by transduction of cells with serially diluted virus in the presence of polybrene (4 μg/mL, Sigma) for 6 h followed by puromycin (Life Technologies) selection 48 h post-infection. 

### 4.6. Plasmid Constructs 

pCW57-FOXM1b (Addgene #68811) and pCW57-FOXM1c (Addgene #68810) were previously described [25]. pCW57-GFP-2A-MCS (Addgene #71783) and pCW57-MCS1-2A-MCS2 (Addgene #71782) were generated by converting pCW57.1 (Addgene #41393) from Gateway cloning to sticky end cloning by adding a GFP-P2A-multiple cloning site and a multiple cloning site, respectively. pCW57-GFP-P2A-E2F1 (RefSeq NM_005225) was generated by PCR subcloning E2F1 (Addgene #21667) into pCW57-GFP-P2A-MCS. pCW57-CCNE1 (RefSeq NM_001322262) was generated by PCR subcloning CCNE1 (Harvard PlasmID Repository, HSCD00326535, RefSeq NM_001322261) into pCW57-MCS1-2A-MCS2. TLCV2 (Addgene #87360) was generated from LentiCRISPR v2 by modifying it into an all-in-one inducible system; doxycycline induces Cas9-P2A-GFP expression. sgRNA and puromycin expression are driven by constitutive promoters. The following modifications were made to lentiCRISPR v2: the EF1a promoter was removed and replaced with a tight Tet Response Element (TRE) promoter; P2A-puromcycin was removed and replaced with T2A-eGFP; and a EF1a promoter-puromycin-P2A-rtTA cassette was added. TLCV2-RB1 (Addgene #87836) was generated by insertion of the previously validated *RB1* guide RNA (sgRB1-GCTCTGGGTCCTCCTCAGGA, [50]) into TLCV2. The 6X-FOXM1 promoter was subcloned from PGL3-6X-FOXM1 [70] into PGL4 to generate PGL4-6X-FOXM1. The PGL3-FOXM1 promoter (−1.1 kb) reporter construct was previously described [70]. The FOXM1 promoter (−2.4 kb) was subcloned from pFlash-FOXM1 [53] into PGL3 to generate PGL3-FOXM1 (−2.4 kb). pCMV6 plasmid (Origene, Rockville, MD, USA) containing FOXM1 isoform a, b, and c was generated with PCR, restriction enzyme digestion and ligation. pCMV6-FOXM1c DNA binding domain mutant (DBD MT) was generated by site-directed mutagenesis (QuikChange, Agilent Technologies, Santa Clara, CA, USA,) of the FOXM1c DNA binding domain at the following residues, R286A, H267A, and S290A, rendering FOXM1 deficient in DNA binding [64]. Primers were designed using QuikChange Primer Design software (Agilent) and are listed in Table S5. pCW57-FOXM1c DBD MT was generated by subcloning FOXM1c DBD MT from pCMV6 into pCW57-MCS1-2A-MCS2 (Addgene #71782). Lentiviral dox inducible shRNA constructs non-silencing (NS, RHS4743), shFOXM1 #1 (V3LHS_396939), and shFOXM1 #2 (V3LHS_396941) were purchased from Dharmacon. All cloning was sequence verified. Plasmids were transfected with Lipofectamine 2000 (Life Technologies), according to the manufacturer’s instructions. 

### 4.7. CRISPR Knockout

CRISPR-mediated FOXM1 knockout was performed as previously described [71]. Briefly, Lipofectamine 2000 (Life Technologies) was used to transfect HEK293T cells with PX458 (Addgene #48138) containing FOXM1 guide RNAs targeting near FOXM1 start and stop codons (462-TGAGAATCAGTGGCCGACGG and 897-CACCGGTCTAAGGGTTCTGAACTG, respectively). The guide RNAs were designed using the Genetic Perturbation Platform Web Portal (https://portals.broadinstitute.org/gpp/public/) and the highest scoring guide RNAs were then empirically tested for their cutting efficiency. Isolation of clonal cell lines was achieved with fluorescence-activated cell sorting (FACS) following by plating of GFP positive cells into 96-well dishes for clonal growth. DNA was isolated from clones with QuickExtract (Epicentre Technologies, Thane, Maharashtra, India) for genotyping and replica plated for clonal expansion. Genotyping was performed by PCR with annealing temperature of 60 °C and 30 cycles for all primer pairs listed in Table S5. Primers were designed with NCBI Primer-BLAST then empirically tested by gradient end-point PCR to optimize the specificity and sensitivity. 

### 4.8. Promoter Activity Luciferase Reporter Assay

The 6X-FOXM1 luciferase reporter assay was performed with pGL4-6X-FOXM1 (firefly) and pRL-TK (renilla, transfection control). The FOXM1 promoter reporter assay was performed with PGL3-FOXM1 promoter (−1.1 kb) and PGL3-FOXM1 promoter (−2.4 kb) and SEAP-PGK (transfection control). Firefly and renilla luciferase activities were measured 24 h after transfection using the Dual-Luciferase Reporter Assay System (Promega, Madison, WI, USA) and GloMax 20/20 luminometer (Promega). Firefly and renilla luciferase activities were expressed in arbitrary units as displayed on the luminometer after a 10 s integration time. SEAP activity was measured 24 h after transfection using the Phospha-Light kit (Thermo Scientific) and POLARstar OPTIMA microplate reader (BMG Labtech, Cary, NC, USA). All transfections were performed in triplicate within each individual experiment. 

### 4.9. RNA Sequencing (RNA-seq) Analysis

RNA was isolated from cell lines according to the method described above. The RNA samples were transported to the UNMC DNA Sequencing Core Facility. RNA samples were analyzed with respect to purity and potential degradation. Purity and concentration were assessed by measurement of the A260/280 ratios using a Nanodrop instrument (Thermo Scientific, Nanodrop Products) instrument and only those samples with values of 1.8 to 2.0 underwent further processing. Potential degradation of the samples was assessed by analysis of 200 ng of the RNA with a Fragment Analyzer (Agilent) and only intact RNA samples were used to generate sequencing libraries. Sequencing libraries were generated by the UNMC NGS Core beginning with 1 µg of total RNA from each sample using the TruSeq V2 RNA sequencing library kit from Illumina following recommended procedures (Illumina, San Diego, CA, USA). Resultant libraries were assessed for size of insert by analysis of an aliquot of each library on a Bioanalyzer instrument (Agilent Technologies, Santa Clara, CA, USA). Each library had a unique indexing identifier barcode allowing the individual libraries to be multiplexed together for efficient sequencing. Multiplexed libraries (12 samples per pool) were sequenced across 2 lanes of the HiSeq 2500 DNA Analyzer (Illumina) to generate a total of approximately 20 million 75 bp single reads for each sample. During sequencing, the quality was continually monitored regarding cluster number and fluorescence intensity and percentages of reads passing filter with a Q30 score. Following sequencing, samples were demultiplexed to produce FASTQ files. The UNMC Epigenomics Core Facility processed the resulting sequence files based on the following steps. Adaptor sequences and low quality (Phred score: 20) ends were trimmed from sequences using the Trim Galore software package (http://www.bioinformatics.babraham.ac.uk/projects/trim_galore/). Resulting FASTQ files were aligned to the human genome (NCBI37/hg19) using the software TopHat (v2.0.8) (http://ccb.jhu.edu/software/tophat/index.shtml). The software Cufflinks (v2.1.1) http://cole-trapnell-lab.github.io/cufflinks/ was used to estimate the expression values, and Cuffdiff (v2.1.1) was used to determine differential expression. Venn diagrams were generated with Venn Diagrams software (Bioinformatics & Evolutionary Genomics-Ghent University http://bioinformatics.psb.ugent.be/webtools/Venn/). 

### 4.10. Statistical Analyses

Student’s *t*-test was used to compare differences between means between two groups. Mann-Whitney test was used to compare differences between medians between two groups. One-way analysis of variance (ANOVA) with a post-test for linear tend was used to compare two or more groups. For all analyses, significance was inferred at *p* < 0.05 and *p* values were two-sided. Graphpad Prism statistical software (GraphPad Software, San Diego, CA, USA) was used. 

### 4.11. Data Deposit

RNA sequencing data is deposited in Gene Expression Omnibus (GEO) (GSE126564).

## 5. Conclusions

FOXM1 is widely overexpressed in human cancer, where its expression is associated with genomic instability. Three major mechanisms driving increased FOXM1 expression in cancer are FOXM1 gene amplification, Tp53 mutation, and Rb-E2F deregulation. FOXM1c is the highest expressed isoform in both normal tissues and cancer. FOXM1b and FOXM1c are transcriptionally active, while FOXM1a is not. 

## Supplementary Materials

The following are available online at https://www.mdpi.com/2072-6694/11/2/251/s1, Figure S1: FOXM1 amplifications + copy number gains in TCGA cancer types, Figure S2: FOXM1 copy number correlations with FOXM1 mRNA and protein expression in CCLE cancer cell lines, Figure S3: FOXM1 expression in TCGA aneuploidy clusters, Figure S4: FOXM1 isoform expression in human cancer cell lines, Figure S5: FOXM1 isoform transcriptional activity in FT282 cells, Figure S6: FOXM1 promoter activity and endogenous mRNA expression following FOXM1 knockout or exogenous FOXM1 overexpression in human cell lines, Table S1: Summary of TCGA cancer types, Table S2: FOXM1 copy number, mRNA, and protein expression in CCLE cell lines, Table S3: RNA-seq differential expression analysis related to Figure 9C–F, Table S4: Lists of genes in Venn Diagram related to Figure 9E, Table S5: Lists of genes in Venn Diagram related to Figure 9F, Table S6: Lists of primers and sequences.
