# Supplementary material for: Pan-Cancer Analyses Reveal Genomic Features of FOXM1 Overexpression in Cancer
Source: Cancers (Basel). 2019 Feb 21;11(2):251. doi: 10.3390/cancers11020251 (PMC6406812; doi:10.3390/cancers11020251)
Supplement: Supplementary file 1 [file cancers-11-00251-s001.pdf]

Supplementary Materials: Pan-Cancer Analyses Reveal Genomic Features of FOXM1 Overexpression in Cancer

Carter J. Barger, Connor Branick, Linda Chee and Adam R. Karpf

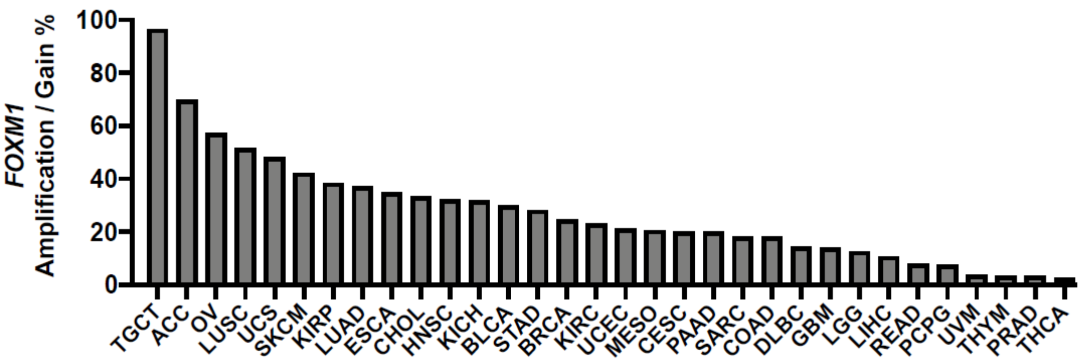

Figure S1. *FOXM1* amplifications + copy number gains in The Cancer Genome Atlas (TCGA) cancer types. *FOXM1* amplification and gain combined frequency in TCGA cancer types as determined by Genomic Identification of Significant Targets in Cancer (GISTIC). Cancer types are ranked by the combined frequency of *FOXM1* amplifications and gains.

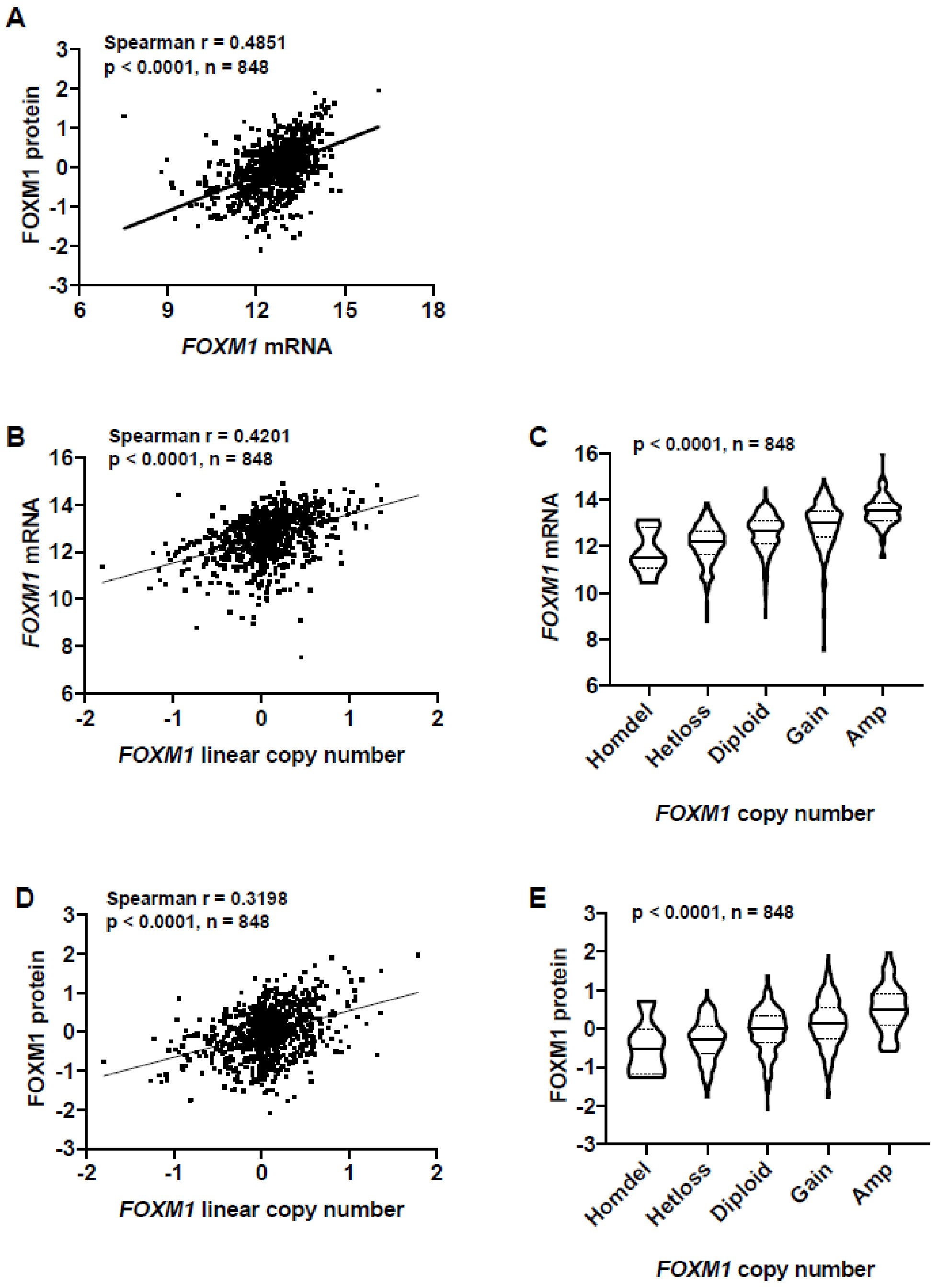

Figure S2. *FOXM1* copy number correlations with FOXM1 mRNA and protein expression in CCLE cancer cell lines. (**A**) *FOXM1* mRNA expression (RPKM) correlation with FOXM1 protein expression (RPPA) across CCLE cancer cell lines. (**B**) *FOXM1* mRNA expression (RPKM) compared to *FOXM1* linear copy number values across CCLE cancer cell lines. (**C**) *FOXM1* mRNA expression (RPKM) compared to *FOXM1* copy number (GISTIC) across CCLE cancer cell lines. The *p* value for ANOVA with post-test for linear trend is shown. Sample lines represent medians and quartiles. (**D**) FOXM1 protein expression (RPPA) correlated with *FOXM1* linear copy number values across CCLE cancer cell lines. (**E**) FOXM1 protein expression (RPPA) compared to *FOXM1* copy number across CCLE cancer cell lines. The *p* value for ANOVA with post-test for linear trend is shown. Sample lines represent medians and quartiles.

A

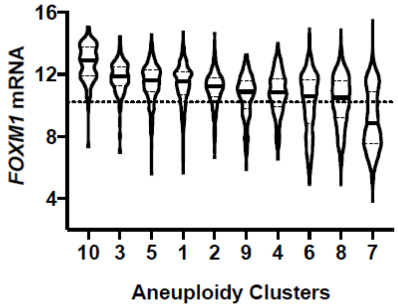

B

Cluster / Aneuploidy	n	Enriched TCGA Cancer Types
10 / mod	144	TCGT
3 / high	258	LUSC, ESCA, HNSC
5 / high	651	CESC, UCS, OV, high-copy UCEC
1 / high	1274	CESC, UCS, OV, high-copy UCEC
2 / high	322	COAD, READ, STAD, ESCA (CIN)
9 / mod	277	GBM
4 / high	329	
6 / mod	998	
8 / mod	1621	Low-copy UCEC
7 / low	3370	THCA, PRAD, THYM, BRCA (Luminal)

C

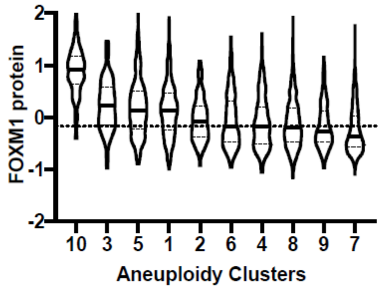

D

Cluster / Aneuploidy	n	Enriched TCGA Cancer Types
10 / mod	106	TCGT
3 / high	106	LUSC, ESCA, HNSC
5 / high	316	CESC, UCS, OV, high-copy UCEC
1 / high	626	CESC, UCS, OV, high-copy UCEC
2 / high	93	COAD, READ, STAD, ESCA (CIN)
6 / mod	570	
4 / high	177	
8 / mod	866	Low-copy UCEC
9 / mod	137	GBM
7 / low	1794	THCA, PRAD, THYM, BRCA (Luminal)

Figure S3. FOXM1 expression in TCGA aneuploidy clusters. (**A**) *FOXM1* mRNA expression (RNA-seq RSEM, log2(norm count + 1)) by aneuploidy clusters and ranked by median *FOXM1* mRNA expression**.** Dotted line across the graph represents median expression value for all primary tumors with aneuploidy cluster group. (**B**) Aneuploidy clusters with degree of aneuploidy showing sample size (*n*) and enriched TCGA cancer types for Panel A. Aneuploidy scores reflect the overall aneuploidy burden, and the range varies across tumor types. Most aneuploidy groups represent a mix of tumor types; however, some represent distinct cancer types and those are listed. (**C**) FOXM1 protein expression (RPPA, pan-can normalized) by aneuploidy clusters and ranked by median FOXM1 protein expression**.** Dotted line across the graph represents median expression value for all primary tumors with aneuploidy cluster group. (**D**) Aneuploidy clusters with degree of aneuploidy showing sample size (*n*) and enriched TCGA cancer types for Panel C.

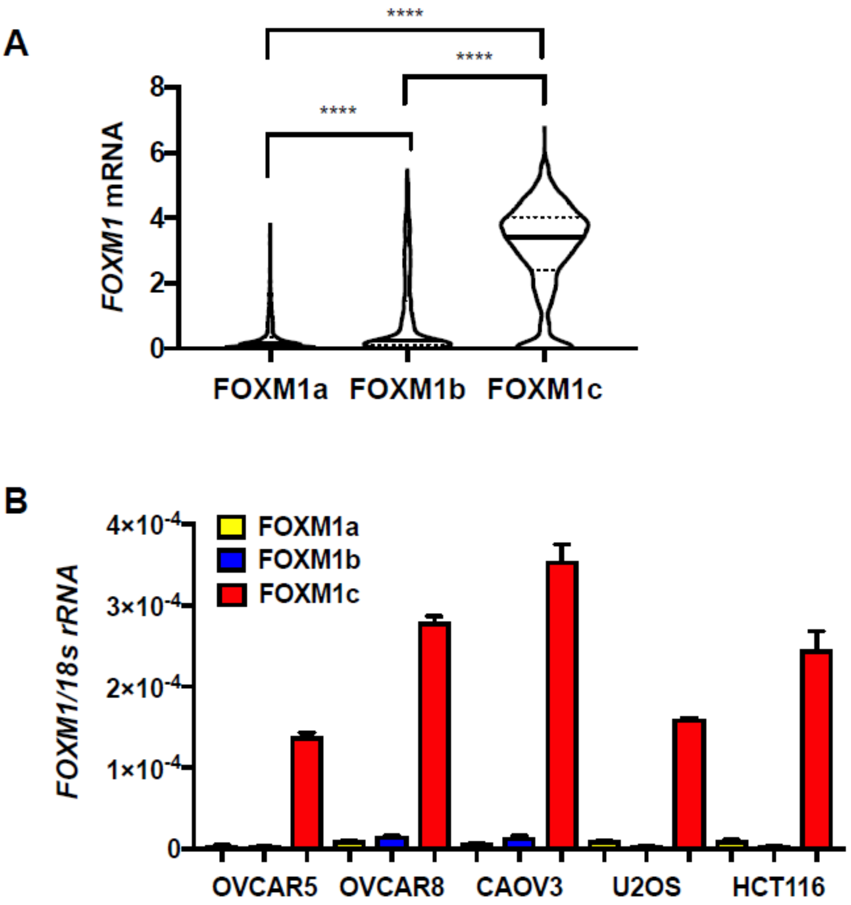

Figure S4. *FOXM1* isoform expression in human cancer cell lines (**A**) *FOXM1* isoform expression (RNA-seq RSEM, log2(FPKM + 1) in CCLE human cancer cell lines (n = 933). (**B**) *FOXM1* isoform expression (qPCR) in a panel of human cancer cell lines. Mann-Whitney test p values are shown. p value designation: **** < 0.0001.

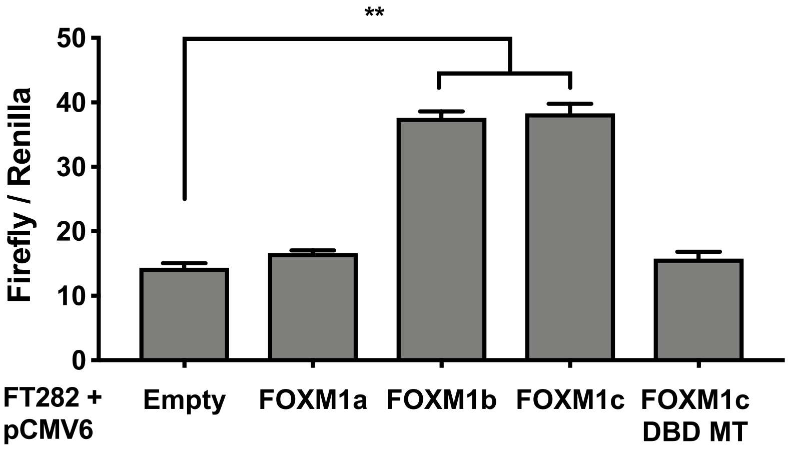

Figure S5. FOXM1 isoform transcriptional activity in FT282 cells. 6X-FOXM1 reporter assay in FT282 cells transiently transfected with FOXM1 isoforms. Student's t test p values are shown. p value designation: ** < 0.01.

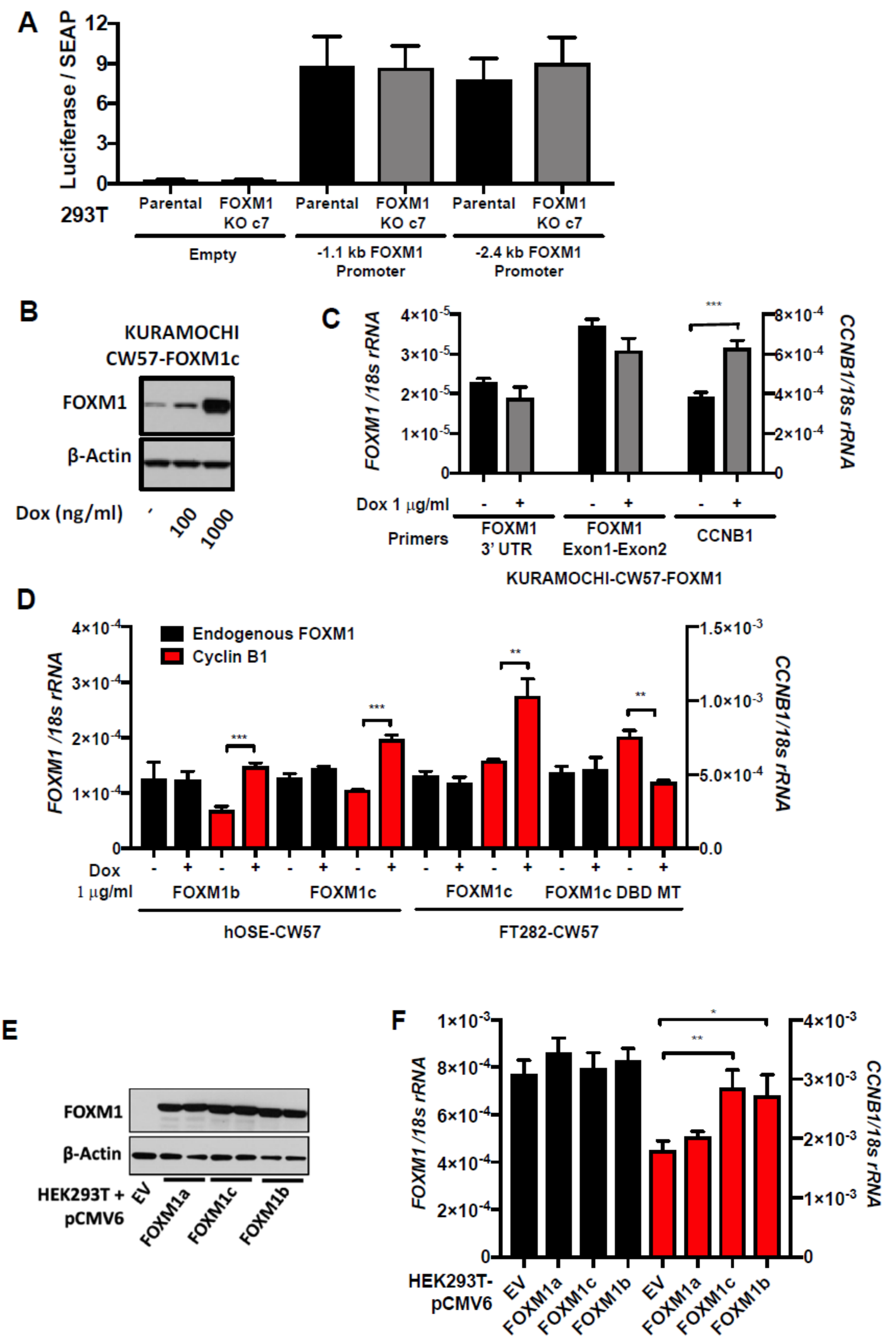

Figure S6. FOXM1 promoter activity and endogenous mRNA expression following FOXM1 knockout or exogenous FOXM1 overexpression in human cell lines. (**A**) FOXM1 promoter activity from two different FOXM1 promoter constructs of varying lengths in HEK293T *FOXM1* knockout cells compared to HEK293T parental cells. Firefly luciferase activity was normalized to the transfection control, secreted embryonic alkaline phosphatase (SEAP). (**B**) FOXM1 protein expression (Western blot) in KURAMOCHI cells engineered for stable dox inducible FOXM1c expression and used in Figure S6C. (**C**) *FOXM1* endogenous (UTR and Exon1/2 primers) and *CCNB1* mRNA expression (RT-qPCR) in KURAMOCHI cells engineered for stable dox inducible FOXM1 expression. (**D**) *FOXM1* endogenous (Exon1/2 primers) and *CCNB1* mRNA expression (RT-qPCR) in cell lines engineered for

stable dox inducible FOXM1 expression (hOSE and FT282, FOXM1 isoforms indicated). (**E**–**F**) Transient FOXM1 expression (HEK293T, FOXM1 isoforms indicated). (**E**) FOXM1 Western blot. (**F**) *FOXM1* endogenous (Exon1/2 primers) and *CCNB1* mRNA expression (RT-qPCR). All time points represent 72 h dox treatment or 72 h post transfection. Bars represent mean ± sd. Student's t test p values are shown. p value designation: *** < 0.001, ** < 0.01, * < 0.05.

Table S1. Summary of TCGA cancer types. TCGA cancer types and sample size. Samples consist of primary tumors and those with overlapping copy number, RNA-seq and RPPA data.

TCGA Cancer Abbreviation	TCGA Cancer Type	Sample Number
ACC	Adrenocortical carcinoma	45
BLCA	Bladder Urothelial Carcinoma	335
LGG	Brain Lower Grade Glioma	417
BRCA	Breast invasive carcinoma	854
CESC	Cervical squamous cell carcinoma and endocervical adenocarcinoma	160
CHOL	Cholangiocarcinoma	30
COAD	Colon adenocarcinoma	235
ESCA	Esophageal carcinoma	123
GBM	Glioblastoma multiforme	63
HNSC	Head and Neck squamous cell carcinoma	331
KICH	Kidney Chromophobe	63
KIRC	Kidney renal clear cell carcinoma	434
KIRP	Kidney renal papillary cell carcinoma	205
LIHC	Liver hepatocellular carcinoma	174
LUAD	Lung adenocarcinoma	354
LUSC	Lung squamous cell carcinoma	319
DLBC	Lymphoid Neoplasm Diffuse Large B-cell Lymphoma	32
MESO	Mesothelioma	61
OV	Ovarian serous cystadenocarcinoma	289
PAAD	Pancreatic adenocarcinoma	97
PCPG	Pheochromocytoma and Paraganglioma	77
PRAD	Prostate adenocarcinoma	343
READ	Rectum adenocarcinoma	71
SARC	Sarcoma	215
SKCM	Skin Cutaneous Melanoma	254
STAD	Stomach adenocarcinoma	334
TGCT	Testicular Germ Cell Tumors	117
THYM	Thymoma	86
THCA	Thyroid carcinoma	362
UCS	Uterine Carcinosarcoma	47
UCEC	Uterine Corpus Endometrial Carcinoma	108
UVM	Uveal Melanoma	12

Table 2. FOXM1 copy number, mRNA, and protein expression in CCLE cell lines. Cell lines are sorted by GISTIC FOXM1 copy number, from homozygous deletion to amplification.

Primary Cell Line Name	FOXM1 Copy Number (Linear)	FOXM1 Copy Number (GISTIC)	FOXM1 mRNA (RNA-seq, RPKM)	FOXM1 Protein (RPPA)
JM1	−0.2559	−2	13.14	0.7183
MHH-CALL-4	−1.029	−2	11.66	−0.2459
HOS	−0.1398	−2	12.75	−0.2479
WM-983B	−1.798	−2	11.38	−0.756
U266B1	−1.161	−2	11.24	−1.125
EFO-21	−1.264	−2	10.45	−1.257
NCI-H1341	−0.2227	−1	13.09	1.003
ECC10	−0.2573	−1	12.61	0.7316
GB-1	−0.0476	−1	13.33	0.6609
COR-L95	−0.1046	−1	13.45	0.5767
SK-MES-1	−0.2962	−1	12.1	0.576
NCI-H1623	−0.4061	−1	13.22	0.5749
MDA-MB-231	−0.1913	−1	13.01	0.5628
TALL-1	−0.4106	−1	11.88	0.5605
LN-229	−0.0487	−1	13.87	0.5557
SNU-449	−0.2438	−1	12.43	0.5295
HT-1376	−0.4037	−1	12.66	0.5222

HEC-6	−0.1934	−1	13.11	0.4906
HMCB	−0.2018	−1	13.47	0.4824
NCI-H1373	−0.4106	−1	12.89	0.4506
K-562	−0.417	−1	11.31	0.4402
KS-1	−0.2191	−1	12.68	0.4388
HD-MY-Z	−0.2366	−1	12.79	0.4144
NCI-H661	−0.3566	−1	11.65	0.3967
HCC1143	−0.2893	−1	12.45	0.3911
KALS-1	−0.0413	−1	12.84	0.3351
GSS	−0.4134	−1	12.36	0.3281
HARA	−0.4283	−1	12.76	0.3243
NCI-H660	−0.5523	−1	12.1	0.3201
NCI-H2196	−0.703	−1	12.45	0.3182
SNU-16	−0.9578	−1	11.97	0.3052
NCI-H1581	−0.4651	−1	12.24	0.2718
8505C	−0.1176	−1	12.89	0.2605
5637	−0.0657	−1	13.59	0.2297
MDA-MB-468	−0.4185	−1	12.16	0.2253
OE33	−0.0722	−1	12.55	0.2102
NCI-H1650	−0.0515	−1	12.98	0.1848
NCI-H2009	−0.2252	−1	12.93	0.169
KPL-1	−0.2261	−1	12.13	0.1551
UACC-257	−0.0616	−1	12.53	0.141
COLO-800	−0.3582	−1	12.62	0.1348
COLO 668	−0.0877	−1	12.52	0.1227
PC-3	−0.5567	−1	12.32	0.1224
EFM-192A	−0.5382	−1	12.7	0.1058
TCCSUP	−0.0725	−1	12.19	0.0931
NCI-H1734	−0.3099	−1	13.27	0.08294
DU 145	−0.3643	−1	11.76	0.04822
EJM	−0.5572	−1	12.28	0.04813
KYO-1	−0.8608	−1	10.38	0.04214
SK-MEL-30	−0.1354	−1	13.43	0.004989
Hs 944.T	−0.3945	−1	13.23	0.002686
TE-4	−0.2699	−1	11.69	−0.00655
NCI-H28	0.0016	−1	12.67	−0.01536
NCI-H358	−0.7272	−1	12.46	−0.02512
L3.3	−0.0943	−1	11.71	−0.03145
SK-CO-1	−0.2214	−1	11.8	−0.04669
M059K	−0.376	−1	12.96	−0.05771
NCI-H1975	−0.3994	−1	12.76	−0.05828
NCI-H1437	−0.5008	−1	11.52	−0.06358
GMS-10	−0.5155	−1	12.83	−0.0707
NCI-H1793	−0.7601	−1	12.34	−0.08221
LP-1	−0.7263	−1	8.771	−0.09862
NCI-H2087	−0.3788	−1	12.43	−0.1009
SUP-T11	−0.8246	−1	12.31	−0.1016
SW 1783	−0.2015	−1	13.03	−0.1123
HuH28	−0.2426	−1	12.62	−0.115
PA-TU-8988S	−0.6317	−1	12.62	−0.1174
RH-41	−0.5606	−1	12.92	−0.128
MDST8	−0.4282	−1	13.87	−0.1349
T98G	−0.058	−1	12.54	−0.136
RERF-LC-MS	−0.1796	−1	12.16	−0.1398
SCLC-21H	−0.8719	−1	11.54	−0.1414
SNU-5	−0.1819	−1	12.94	−0.1476
YAPC	−0.6467	−1	11.44	−0.1575
TCC-PAN2	−0.5364	−1	11.07	−0.1623
CAS-1	−0.1373	−1	12.55	−0.1675
P12-ICHIKAWA	−0.88	−1	12.52	−0.1707
THP-1	−1.224	−1	11.95	−0.1849
LN-18	−0.3031	−1	13.09	−0.186

ONS-76	−0.2167	−1	11.38	−0.1894
NCI-H2227	−0.3177	−1	12.27	−0.2057
OVCAR-4	−0.1156	−1	11.96	−0.2148
NCI-H1781	−0.2913	−1	13.29	−0.232
MKN1	−0.5728	−1	12.59	−0.2343
NCI-H2172	−0.5688	−1	12.81	−0.2451
NCI-H889	−0.4476	−1	11.76	−0.2456
WM-266-4	−0.6034	−1	10.24	−0.2602
S-117	−0.1931	−1	12.25	−0.2708
HCC38	−0.5804	−1	12.91	−0.2765
SNU-201	−0.0331	−1	12.46	−0.2988
OVSAHO	−0.414	−1	11.28	−0.2988
TE-14	−0.5014	−1	10.98	−0.2993
PE/CA-PJ34 (clone C12)	−0.578	−1	12.02	−0.3051
SCC-9	−0.6126	−1	11.68	−0.3071
FTC-133	−0.3404	−1	13.07	−0.3313
SNU-1105	−0.2165	−1	13.01	−0.3419
HCC-44	−0.085	−1	11	−0.343
SNU-668	−0.4444	−1	12.13	−0.3452
MCF7	−0.3487	−1	12	−0.3567
DMS 273	−0.3343	−1	12.4	−0.3711
HCC202	−0.3707	−1	12.54	−0.3716
PK-59	−0.8966	−1	11.02	−0.3747
OAW42	−0.2744	−1	12.72	−0.3933
RPMI-7951	−0.2576	−1	11.86	−0.4107
KLE	−0.3269	−1	12.11	−0.4161
NCI-H596	−0.5133	−1	12.2	−0.4266
GI-1	−0.1537	−1	12.83	−0.427
KURAMOCHI	−0.334	−1	10.27	−0.4339
KMS-28BM	−0.9761	−1	11.85	−0.4494
JHUEM-3	−0.3553	−1	12.04	−0.4501
SNU-213	−0.4614	−1	11.44	−0.4529
MOLM-6	−0.2093	−1	11.8	−0.4557
MDA-MB-157	−1.056	−1	11.91	−0.4657
NCI-H1666	−0.4756	−1	10.69	−0.4846
CFPAC-1	−0.7384	−1	10.92	−0.515
MKN7	−0.6549	−1	11.41	−0.5192
VM-CUB1	−0.2131	−1	9.175	−0.5192
A-253	−0.4107	−1	12.54	−0.5216
SK-OV-3	−0.9342	−1	12.02	−0.5266
SNU-423	−0.4382	−1	12.47	−0.5396
PA-TU-8988T	−0.5514	−1	13.54	−0.5501
SW403	−0.5659	−1	10.67	−0.5656
SKM-1	−0.8694	−1	12.15	−0.59
REC-1	−0.6277	−1	11.5	−0.6272
MDA-MB-415	−0.7052	−1	10.65	−0.6293
UACC-812	−0.3497	−1	9.455	−0.6427
OV7	−0.8403	−1	12.19	−0.6494
NCI-H2342	−0.5628	−1	11.99	−0.6503
NCI-H2126	−0.1798	−1	11.4	−0.6538
42-MG-BA	−0.3497	−1	12.92	−0.6612
PANC-1	−0.3975	−1	13.25	−0.6678
NCI-H1563	−0.3142	−1	12.37	−0.7139
JHH-2	−0.8264	−1	11.26	−0.7156
U-251 MG	−0.4152	−1	11.74	−0.7214
YD-8	−0.1488	−1	10.7	−0.7561
UM-UC-1	−0.3661	−1	10.64	−0.7625
SNU-475	−0.5704	−1	12.08	−0.7803
Capan-1	−0.4115	−1	11.65	−0.8103
SHP-77	−0.8669	−1	12.17	−0.8673
CAMA-1	−0.9042	−1	12.17	−0.8718

HPAF-II	−0.4037	−1	11.45	−0.8811
TC-71	−0.8978	−1	12.19	−0.9154
SW-1710	−0.3451	−1	10.57	−0.9178
Hs 766T	−0.2887	−1	12.13	−0.9302
EM-2	−0.3726	−1	11.72	−0.931
SNU-216	−0.5394	−1	11.69	−0.9328
NCI-H508	−0.1057	−1	10.16	−0.953
OVKATE	−0.8895	−1	11.2	−0.962
NCI-H2122	−0.3132	−1	11.39	−0.9898
NCI-H1651	−0.4748	−1	12.61	−1.023
HCC4006	−0.2546	−1	12.46	−1.043
COR-L311	−0.6728	−1	11.93	−1.065
NCI-H1693	−0.3835	−1	11.99	−1.08
MeWo	−1.086	−1	12.37	−1.093
WM-88	−0.2742	−1	11.23	−1.174
Hs 578T	−0.3064	−1	12.04	−1.215
Kasumi-6	−1.081	−1	10.62	−1.231
SNU-489	−0.2268	−1	11.62	−1.279
KMS-21BM	−1.142	−1	10.79	−1.288
NCI-H522	−0.2672	−1	11.81	−1.292
NCC-StC-K140	−0.1073	−1	10.71	−1.294
SNU-1077	−0.1122	−1	10.67	−1.322
8305C	−0.4195	−1	12.36	−1.418
CAL-12T	−0.3334	−1	12.28	−1.499
MCAS	−0.2026	−1	12.06	−1.524
A172	−0.8024	−1	12.41	−1.746
MONO-MAC-6	−0.8135	−1	11.69	−1.769
NCI-H1155	−0.0739	0	14.05	1.378
SNU-410	0.1766	0	13.92	1.327
SEM	0.0757	0	12.59	1.321
KM12	0.042	0	11.58	1.173
HEC-59	0.0583	0	13.14	1.064
NCI-H1105	0.3345	0	13.55	0.9938
JHH-7	0.0427	0	13.29	0.9887
LU99	0.2004	0	13.37	0.9761
KNS-62	0.2468	0	12.06	0.9705
NALM-19	0.0308	0	13.83	0.9601
NCI-H1618	0.0752	0	13.71	0.9471
NU-DUL-1	0.0292	0	12.68	0.9217
HGC-27	0.079	0	13.64	0.9169
DMS 79	0.2752	0	13.27	0.8795
NCI-H747	0.0558	0	12.42	0.8767
NCI-H716	0.1512	0	13.33	0.8648
MC116	−0.0271	0	12.66	0.8556
RS4;11	0.064	0	10.29	0.8303
Panc 10.05	0.1874	0	13.73	0.79
LXF-289	0.037	0	11.32	0.7679
YKG1	−0.0274	0	12.63	0.7394
KOPN-8	0.0043	0	13.52	0.7262
SNU-1	−0.02	0	13.52	0.7224
DAN-G	0.1158	0	13.14	0.7014
LMSU	−0.198	0	13.21	0.6991
BL-70	0.0016	0	12.93	0.6963
NCI-H69	0.4018	0	13.49	0.6854
CCF-STTG1	−0.0245	0	13.65	0.6786
KCL-22	−0.0783	0	12.95	0.6707
J82	0.041	0	13.78	0.6667
SNU-C2A	0.0225	0	13	0.6611
SW48	0.013	0	10.59	0.6351
MEL-JUSO	−0.1334	0	13.35	0.6348
SNU-324	0	0	12.91	0.6294
ESS-1	0.0087	0	13.27	0.6036

NCI-H1435	0.1625	0	13.15	0.6009
SK-UT-1	0.0311	0	13.6	0.6008
SNU-601	−0.1157	0	13.23	0.5993
GRANTA-519	0.0974	0	11.95	0.5881
EN	0.0277	0	13.04	0.5795
NCI-H1838	0.0289	0	13.54	0.5744
HCC70	−0.0425	0	13.18	0.5661
DMS 153	0.1481	0	13.61	0.5658
CCK-81	0.1245	0	12.68	0.5643
CAL-62	−0.0082	0	13.15	0.5526
KMRC-20	0.0953	0	13.18	0.5414
786-O	0.0893	0	13.16	0.5409
SNU-478	0.0904	0	12.92	0.5387
BC-3C	−0.0585	0	12.96	0.5378
PF-382	0.015	0	13.55	0.5308
SK-N-BE (2)	0.0677	0	13.55	0.5233
NCI-H2228	0.1054	0	12.68	0.5192
HuT 78	0.135	0	12.85	0.5182
KELLY	−0.0115	0	12.68	0.5112
NALM-1	0.0659	0	12.7	0.4972
ACC-MESO-1	−0.0027	0	12.33	0.4944
AN3 CA	−0.0936	0	13.54	0.4918
NCI-H211	0.1525	0	13.74	0.4859
RPMI 8226	0.1214	0	12.26	0.4837
MHH-CALL-2	−0.0309	0	13.2	0.4806
COLO 741	0.0281	0	12.94	0.479
NCI-H209	0.0799	0	14.31	0.4664
HEC-265	0.0342	0	13.11	0.4649
RERF-LC-AI	−0.1588	0	13.48	0.4622
SNU-1196	−0.2346	0	13.47	0.462
NCI-H1436	0.0134	0	13.08	0.4582
RCH-ACV	−0.011	0	13.73	0.4571
HCC-15	0.2507	0	13.48	0.448
KP-N-YN	0.0754	0	13.18	0.4468
IGR-37	0.1974	0	14.51	0.4458
CHP-212	0.0029	0	12.9	0.4453
HPB-ALL	0.0967	0	13.68	0.4345
SBC-5	0.0491	0	12.96	0.4293
CAL-51	−0.0187	0	12.72	0.4278
PEER	0.0457	0	13.75	0.422
SK-MEL-3	−0.0182	0	12.82	0.4188
BDCM	0.0063	0	12.46	0.4156
MDA-MB-453	−0.0671	0	12.87	0.4146
KHM-1B	−0.2344	0	11.87	0.4105
KP-N-RT-BM-1	0.0184	0	12.88	0.4058
YD-15	0.0176	0	11.74	0.4052
COR-L24	0.1896	0	12.81	0.4007
IM95	−0.0649	0	12.87	0.3981
MHH-NB-11	−0.0743	0	12.19	0.3945
SNU-C1	−0.0753	0	11.99	0.3943
YH-13	0.1647	0	11.33	0.3873
CHP-126	−0.0255	0	12.88	0.3795
PSN1	0.0643	0	12.12	0.3793
ECC12	−0.1513	0	13.01	0.3742
TE-11	0.0963	0	13.23	0.366
SNU-245	−0.0678	0	12.22	0.354
OCI-AML3	−0.0397	0	12.24	0.3529
MEL-HO	−0.0935	0	12.93	0.3505
KMS-26	−0.1466	0	12.56	0.3499
SNU-C4	−0.0101	0	12.79	0.3475
COLO 684	−0.1465	0	13.3	0.3465
KMS-11	−0.0462	0	12.11	0.3458

GDM-1	0.0332	0	12.56	0.3415
NCI-H1568	−0.1429	0	12.85	0.336
NALM-6	0.0546	0	12.73	0.3357
KASUMI-2	0.0679	0	12.36	0.335
AML-193	−0.0937	0	11.53	0.3214
RCM-1	−0.0402	0	11.7	0.3194
SW 780	0.1017	0	12.93	0.3183
G-361	−0.0481	0	13.05	0.3127
Hs 611.T	−0.0398	0	12.66	0.3115
BL-41	−0.0261	0	12.71	0.3069
SNU-407	−0.0209	0	12.52	0.3055
NH-6	0.0089	0	13.22	0.3036
L-540	0.1148	0	11.9	0.3008
MHH-CALL-3	0.0013	0	13.44	0.2938
TE-9	0.0717	0	12.71	0.2935
Hs 936.T	−0.0087	0	13.09	0.2848
NCI-H441	0.1547	0	13.04	0.2669
NCI-H524	−0.1324	0	12.17	0.2628
SIMA	0.0249	0	12.73	0.2618
ST486	−0.0794	0	13.03	0.2572
VMRC-RCZ	0.1297	0	12.32	0.25
DND-41	0.0757	0	12.2	0.2475
A2058	−0.1115	0	13.13	0.2415
SK-N-MC	0.0862	0	13.42	0.2401
Hs 939.T	0.0166	0	13.49	0.2391
SNU-899	−0.014	0	11.87	0.2354
OCI-AML2	−0.062	0	12.56	0.2269
COLO 792	0.1606	0	13.41	0.2193
MPP 89	−0.1812	0	13.01	0.2172
LU65	−0.1442	0	12.42	0.2169
MM1-S	0.0801	0	12.93	0.2146
NCI-H1355	−0.2016	0	13.39	0.2139
EB1	−0.0033	0	12.58	0.2114
HH	0.0347	0	12.69	0.2113
SK-MM-2	0.0868	0	12.72	0.2107
HCT-15	0.0173	0	13.43	0.2096
D283 Med	0.0749	0	13.07	0.2075
SNU-398	0.193	0	13.72	0.206
SK-N-AS	0.0078	0	12.97	0.2029
D341 Med	−0.0875	0	8.948	0.1996
ChaGo-K-1	−0.0738	0	12.47	0.1958
HT	0.0991	0	13.02	0.1908
Hs 852.T	0.0055	0	13.39	0.1841
WM-793	−0.1256	0	12.24	0.1834
KARPAS-299	0.0814	0	11.94	0.1769
HCC2935	0.0171	0	12.79	0.1709
TOV-21G	−0.0776	0	11.77	0.1687
KG-1	0.0347	0	12.69	0.1684
Hs 683	0.0735	0	13.74	0.1632
VMRC-RCW	−0.0658	0	10.84	0.1631
GA-10	0.0162	0	12.99	0.1607
MG-63	−0.0689	0	13.34	0.1575
LCLC-103H	0.0764	0	12.28	0.1513
SNU-61	0.1803	0	13.33	0.151
CAL 27	0.0391	0	11.34	0.1497
KP-N-SI9s	−0.2025	0	12.16	0.1459
SNU-308	0.066	0	12.92	0.1377
GP2d	0.002	0	12.9	0.1357
RI-1	−0.0993	0	13.72	0.1328
LOU-NH91	−0.0944	0	12.84	0.1216
HEC-1-B	0.1507	0	13.21	0.1105
Hs 695T	−0.2374	0	13.33	0.1098

RT-112	−0.0588	0	12.53	0.1042
CL-34	0.0396	0	12.51	0.09789
TE 441.T	−0.0267	0	11.96	0.09782
SK-N-SH	−0.087	0	13.14	0.09736
SU-DHL-10	0.0333	0	12.92	0.09642
JHUEM-2	−0.0942	0	12.87	0.09389
OV56	0.0962	0	10.74	0.09097
K029AX	0.1019	0	12.24	0.09049
SUP-T1	−0.0294	0	12.74	0.09048
MEC-1	0.0264	0	12.86	0.07226
SNU-719	−0.0751	0	10.95	0.07105
ABC-1	−0.2587	0	13.03	0.07104
CL-11	0.0872	0	12.9	0.06522
HCC1419	0.1364	0	12.91	0.06454
SU-DHL-6	−0.0504	0	12.55	0.06231
SUP-B15	0.0406	0	12.81	0.06083
AGS	−0.0632	0	13.78	0.04717
HUP-T4	−0.0634	0	12.14	0.04663
MOLT-16	0.0117	0	12.62	0.03867
MFE-296	0.0165	0	14.29	0.03737
SW 1990	0.2369	0	13.75	0.03547
HuNS1	−0.0122	0	12.76	0.03485
CAL-148	0.0056	0	13.68	0.03176
P3HR-1	0.0056	0	13.04	0.02941
SF126	0.0344	0	13.29	0.02807
Capan-2	0.3073	0	13.28	0.02489
SW837	0.1361	0	11.85	0.0216
SH-4	−0.1468	0	12.84	0.01867
DMS 454	0.1668	0	13.48	0.01619
NCI-H841	0.1045	0	14.35	0.01605
NCI-H2066	−0.1312	0	12.31	0.0119
KNS-42	−0.0121	0	13.49	0.01162
RERF-GC-1B	0.1798	0	12.58	0.008544
Set-2	0.0403	0	12.28	0.00744
L-363	0.0011	0	12.45	0.002566
DV-90	−0.0195	0	12.22	−0.004821
KYSE-520	0.1385	0	12.91	−0.009047
SNU-886	0.0504	0	13.6	−0.009294
TF-1	−0.2524	0	12.46	−0.01391
KNS-60	0.0741	0	12.53	−0.01866
A-673	−0.0057	0	13.58	−0.02107
NAMALWA	0.0121	0	12.7	−0.0232
KU812	−0.2728	0	11.38	−0.02458
huH-1	0.0486	0	12.3	−0.02562
SNU-175	−0.0255	0	12.47	−0.02727
NCI-N87	0.2069	0	13.48	−0.03529
BICR 18	0.1561	0	12	−0.04801
HLF	0.1226	0	13.23	−0.05186
DBTRG-05MG	0.0717	0	11.44	−0.05322
SNU-620	−0.112	0	12.7	−0.0581
8-MG-BA	−0.1305	0	12.17	−0.05921
HT-144	0.0604	0	12.84	−0.05972
HT55	0.0686	0	12.16	−0.06062
MOLP-8	0.0124	0	12.96	−0.0625
Loucy	0.1118	0	12.95	−0.06493
MFE-319	−0.0206	0	12.55	−0.06631
HUP-T3	0.2184	0	13.43	−0.07335
KM-H2	0.1024	0	11.89	−0.07978
SF-295	−0.0796	0	13.28	−0.08286
HEC-151	0.0343	0	12.32	−0.0832
HCC-366	−0.0579	0	13.05	−0.08496
NCI-H2347	0.138	0	13.6	−0.08594

SW948	0.1128	0	11.83	−0.08767
LS 180	−0.0332	0	12.23	−0.08962
NUGC-3	−0.2554	0	11.66	−0.1088
RMUG-S	0.2215	0	13.06	−0.1106
HCC1500	0.28	0	11.96	−0.112
NCI-H2405	0.0997	0	13.13	−0.1123
DU4475	0.0315	0	12.32	−0.1123
P31/FUJ	0.0352	0	12.84	−0.1133
NCI-H2286	0.1096	0	14.06	−0.1229
NB-4	−0.1508	0	12.34	−0.135
G-402	−0.0492	0	12.26	−0.1419
Daudi	0.0286	0	12.94	−0.1452
CI-1	−0.0015	0	12.78	−0.153
LAMA-84	0.0064	0	11.3	−0.1549
SW1116	0.1857	0	10.74	−0.1586
A-204	−0.0114	0	12.51	−0.166
EFM-19	0.0775	0	11.98	−0.1694
OCUM-1	−0.1745	0	12.24	−0.1778
SNU-8	0.0313	0	12.3	−0.1806
Hs 729	−0.0582	0	12.93	−0.1821
T-47D	0.1308	0	12.61	−0.1832
HCC1569	−0.0513	0	13.13	−0.1913
LNCaP clone FGC	0.0732	0	12.18	−0.1948
SNU-387	0.0202	0	13.08	−0.2073
GCIY	0.1313	0	12.59	−0.2179
CMK	0.1104	0	12.08	−0.2285
NCI-H929	−0.0015	0	11.29	−0.2289
KYSE-510	0.1031	0	12.47	−0.2294
OCI-M1	0.0256	0	11.45	−0.2308
OVK18	−0.0529	0	13.76	−0.2324
KMS-27	−0.025	0	11.7	−0.2363
23132/87	−0.1018	0	11.97	−0.2414
SNU-520	−0.1479	0	12.82	−0.243
NIH: OVCAR-3	−0.0914	0	11.9	−0.2431
SW 1088	−0.1354	0	12.03	−0.2434
SNU-1197	0.023	0	12.52	−0.2456
BFTC-905	0.1997	0	11.81	−0.246
COLO-783	0.0704	0	12.41	−0.2468
NCI-H1792	−0.0975	0	12.64	−0.2478
HuG1-N	0.1298	0	13.19	−0.2479
PE/CA-PJ49	0.0424	0	13.43	−0.2532
KARPAS-620	0.0394	0	12.3	−0.2559
KO52	−0.0289	0	11.96	−0.2603
TE 617.T	0.0046	0	13.11	−0.2608
KMS-20	0.1684	0	12.52	−0.2611
PE/CA-PJ15	−0.0577	0	11.83	−0.2617
CJM	0.1337	0	12.41	−0.2662
HEC-50B	−0.1487	0	12.28	−0.2689
SNU-840	0.0391	0	13.03	−0.2724
MJ	0.0162	0	13.14	−0.2817
Mino	−0.0118	0	12.77	−0.2924
OC 314	0.0298	0	12.38	−0.2964
FTC-238	−0.0888	0	12.86	−0.2972
MEG-01	−0.111	0	11.67	−0.3013
DEL	−0.1285	0	11.51	−0.3036
LS513	−0.1148	0	11.94	−0.3049
HCC-56	0.0996	0	11.76	−0.3127
MKN74	−0.1132	0	12.18	−0.3176
PE/CA-PJ41 (clone D2)	0.0701	0	12.53	−0.3245
JHUEM-1	0.0176	0	13.39	−0.3278
KMS-12-BM	0.1454	0	13.27	−0.3307

SW1417	0.0153	0	12.77	−0.3345
A-498	−0.1047	0	12.1	−0.3382
AsPC-1	−0.2705	0	10.28	−0.3437
SNU-466	0.0525	0	12.53	−0.3448
C32	−0.0155	0	12.43	−0.3462
SNU-1214	0.1145	0	12.82	−0.3479
ZR-75-30	0.1254	0	11.88	−0.3573
SNU-761	−0.1649	0	11.49	−0.359
NMC-G1	−0.1369	0	13.41	−0.3716
769-P	0.0314	0	12.89	−0.3754
GOS-3	0.1832	0	13.58	−0.3755
CADO-ES1	−0.0552	0	12.1	−0.383
SNU-1040	−0.0034	0	12.74	−0.3905
SCC-4	0.0147	0	12.08	−0.3927
NCI-H292	−0.0022	0	13	−0.4001
HCC-78	−0.0176	0	12.11	−0.4028
SNU-81	−0.0196	0	10.04	−0.4059
GAMG	0.0633	0	13.17	−0.4082
MDA PCa 2b	−0.0008	0	12.28	−0.4253
RD	−0.052	0	13.62	−0.4265
NCI-H2030	−0.0204	0	13.05	−0.4309
TE-1	−0.12	0	13.27	−0.4358
KNS-81	0.0787	0	12.93	−0.4388
HCC2218	0.0386	0	11.72	−0.4452
HEC-251	−0.023	0	12.27	−0.4478
DB	−0.007	0	12.36	−0.4534
F-36P	0.1561	0	12.39	−0.4548
CAL-33	−0.0337	0	11.61	−0.4912
Calu-3	0.0791	0	11.82	−0.5246
MV-4-11	−0.028	0	11.94	−0.5268
JVM-2	−0.0168	0	10.45	−0.5342
M-07e	−0.0033	0	12	−0.5548
JHUEM-7	0.0006	0	11.66	−0.5643
COV434	0.0006	0	12.51	−0.5705
CL-14	0.1816	0	11.76	−0.5716
KMRC-1	0.0833	0	10.66	−0.5778
NCI-H2452	0.2571	0	13.56	−0.5949
SNU-1079	0.1192	0	11.91	−0.5996
SCC-15	−0.0293	0	12.15	−0.6095
A2780	0.0439	0	13.18	−0.619
CML-T1	0.0455	0	12.04	−0.6195
NCI-H2085	−0.1075	0	13.1	−0.6239
OUMS-23	0.1751	0	11.7	−0.6285
Pfeiffer	−0.0584	0	12.5	−0.6329
CW-2	0.0265	0	13.03	−0.6461
COR-L23	−0.1111	0	11.65	−0.6513
IGROV1	0.0034	0	12.26	−0.6627
SK-MEL-5	−0.0621	0	12.77	−0.6731
EOL-1	−0.0567	0	12.18	−0.6754
Hep G2	−0.1272	0	12.22	−0.6849
HEL	0.1374	0	12.26	−0.6967
JL-1	0.0283	0	12.48	−0.7132
TUHR14TKB	0.1468	0	11.54	−0.7182
WM-115	0.0263	0	12.25	−0.733
KMRC-3	0.0344	0	11.19	−0.7342
NOMO-1	0.0145	0	12.19	−0.7358
HT-1080	−0.0079	0	13.17	−0.7367
KE-39	0.1281	0	12.93	−0.7431
TE-15	0.0846	0	13.31	−0.7439
NCI-H838	−0.1925	0	12.43	−0.7444
SNU-1272	0.1068	0	11.75	−0.7535
SNU-626	−0.0847	0	13.14	−0.756

OVISE	0.0364	0	11.58	−0.7595
SNG-M	−0.0128	0	11.03	−0.7668
Hs 294T	0.1121	0	12.7	−0.7701
Caki-1	0.0703	0	10.49	−0.7757
A-375	0.0862	0	13.21	−0.784
ML-1	0.1964	0	12.64	−0.784
HEC-108	0.1324	0	12.2	−0.807
SK-MEL-28	0.0056	0	11.65	−0.8094
TYK-nu	−0.2336	0	13.13	−0.8423
MES-SA	0.0582	0	12.16	−0.8538
RL95-2	−0.0226	0	12.76	−0.8608
NCO2	−0.1716	0	11.24	−0.8976
COLO 829	−0.0033	0	11.48	−0.9059
BICR 22	0.0717	0	9.974	−0.9256
U-87 MG	0.0827	0	12.68	−0.9398
HCT 116	−0.0228	0	12.96	−0.9475
DK-MG	0.1061	0	11.03	−0.9653
A3/KAW	−0.0274	0	9.758	−0.9662
G-401	0.0188	0	12.88	−0.9765
OCI-AML5	−0.0108	0	12.04	−1.006
KASUMI-1	0.0571	0	11.82	−1.016
KARPAS-422	−0.0277	0	11.11	−1.036
KMRC-2	0.0161	0	11.09	−1.097
MOLM-13	−0.1016	0	11.37	−1.102
SK-MEL-24	0.071	0	12.72	−1.113
SW 1353	−0.0182	0	12.25	−1.119
BICR 31	0.0263	0	11.93	−1.165
OCI-LY3	0.04	0	13.39	−1.214
JK-1	−0.0248	0	10.6	−1.215
EHEB	0.008	0	10.72	−1.218
IST-MES1	−0.0911	0	13.16	−1.227
HT-29	−0.0407	0	11.98	−1.236
RMG-I	−0.0825	0	10.93	−1.237
HEC-1-A	−0.0554	0	13.52	−1.243
JHOS-4	−0.0381	0	12.87	−1.251
EFO-27	−0.048	0	12.32	−1.274
B-CPAP	−0.0846	0	9.229	−1.294
MONO-MAC-1	0.0098	0	11.79	−1.367
NCI-H460	−0.3008	0	12.9	−1.485
HL-60	0.0654	0	10.02	−1.492
TT	0.1241	0	12.12	−1.505
NCI-H526	0.0522	0	13.02	−1.55
NCI-H1395	0.0434	0	12.26	−1.572
Panc 05.04	0.1007	0	12.13	−2.088
COR-L88	0.8008	1	13.99	1.903
U-2 OS	0.6141	1	14.3	1.706
639-V	0.4961	1	14.43	1.529
JMSU-1	0.2705	1	14.33	1.471
NCI-H196	0.4427	1	14.35	1.398
PC-14	0.4494	1	13.47	1.365
HCC-95	0.6326	1	14.11	1.357
NUGC-2	1.033	1	14.55	1.314
HuCCT1	0.4556	1	7.516	1.301
NCI-H1694	0.685	1	14.22	1.296
NCI-H650	0.748	1	12.64	1.188
MKN-45	0.6644	1	13.53	1.18
RKN	0.3773	1	13.86	1.134
RERF-LC-Ad1	0.1715	1	13.48	1.107
JIMT-1	0.601	1	13.39	1.096
NCI-H1092	0.512	1	14.1	1.088
IPC-298	0.114	1	12.77	1.056
HCC1599	0.1782	1	12.93	1.03

JHH-6	0.3584	1	13.39	1.027
SNU-C5	0.5361	1	11.85	1
NCI-H520	0.1759	1	13.77	0.9933
C2BBe1	0.4706	1	13.67	0.9854
NCI-H2171	0.5492	1	13.33	0.9841
LUDLU-1	0.2476	1	13.45	0.9831
TT2609-C02	0.3279	1	13.34	0.9718
HCC1395	0.1726	1	14.05	0.9699
KYSE-410	0.2744	1	13.14	0.9581
VCaP	0.2964	1	14.2	0.9473
OPM-2	0.3103	1	12.42	0.9334
NCI-H810	0.6288	1	13.91	0.9266
SW 1271	0.5388	1	13.97	0.915
Toledo	0.3863	1	13.71	0.9062
SK-N-DZ	−0.9394	1	14.42	0.8514
NCI-H1299	0.722	1	14.11	0.8501
KMS-34	0.4083	1	13.21	0.8398
TE-8	0.5689	1	12.48	0.8215
NCI-H1755	0.4957	1	13.92	0.8114
NCI-H146	0.4358	1	13.85	0.7995
MDA-MB-435S	0.2592	1	12.81	0.7973
NCI-H1869	0.3059	1	11.4	0.7961
MOR/CPR	0.2804	1	12.88	0.792
LK-2	0.2332	1	12.81	0.7602
NCI-H2291	0.3729	1	13.27	0.757
KYSE-140	0.2989	1	12.73	0.7546
COLO-320	0.3199	1	13.22	0.7397
AM-38	0.2442	1	13.94	0.7315
YD-38	0.4769	1	11.41	0.7275
SW620	0.3282	1	13.43	0.7166
KYSE-450	0.4513	1	12.96	0.7131
OCI-LY-19	0.5841	1	13.64	0.7095
A4/Fuk	0.5999	1	12.68	0.7073
TOV-112D	0.3345	1	13.4	0.7063
CAL-78	0.1861	1	12.09	0.7052
Li-7	0.5238	1	13.49	0.6971
Calu-6	0.5123	1	13.71	0.6897
MHH-ES-1	0.486	1	13.54	0.6892
U-118 MG	0.2504	1	14.91	0.6525
RH-30	0.2464	1	14.18	0.6459
KATO III	0.1632	1	13.34	0.6433
NCI-H1836	0.3385	1	13	0.6299
SUIT-2	0.4332	1	12.42	0.6059
Panc 03.27	0.6522	1	13.19	0.6011
NCI-H1915	0.1045	1	12.98	0.5575
GCT	0.1478	1	13.44	0.5543
PK-1	0.3841	1	13.54	0.5532
QGP-1	0.3063	1	12.98	0.5343
OE19	0.2029	1	12.67	0.5335
KE-97	0.5493	1	12.98	0.5279
NCI-H647	0.2892	1	13.37	0.5105
NCI-H82	0.3051	1	12.56	0.5084
TGBC11TKB	0.547	1	11.59	0.5061
T3M-4	0.4329	1	12.79	0.4776
YD-10B	0.5431	1	12.14	0.4614
NCI-H1930	0.5004	1	13.51	0.4566
HCC1187	0.447	1	13.91	0.4524
BV-173	0.078	1	12.24	0.448
UACC-893	0.8195	1	12.99	0.4378
NCI-H446	0.3559	1	13.44	0.4371
HuH-7	0.1685	1	13.1	0.4253
MIA PaCa-2	0.4676	1	13	0.3995

NCI-H2029	0.2268	1	13.11	0.3995
SNU-283	0.321	1	13.08	0.3966
RL	0.5182	1	12.98	0.394
KMM-1	0.1373	1	12.99	0.3882
MSTO-211H	0.2378	1	12.65	0.386
NCI-H854	0.2971	1	12.25	0.3847
RPMI-8402	0.2415	1	13.58	0.3796
COLO-679	0.557	1	12.86	0.3792
L-428	0.5138	1	13.33	0.3735
SK-N-FI	0.5785	1	14.07	0.3729
KYSE-70	0.1394	1	11.74	0.3669
LOX IMVI	0.2533	1	13.14	0.3635
CA46	0.0387	1	12.05	0.3475
IMR-32	0.4197	1	13.46	0.3367
HT115	0.4934	1	13.61	0.3328
Panc 04.03	0.2164	1	13.26	0.3313
MDA-MB-134-VI	0.6617	1	11.73	0.3253
Ki-JK	0.2062	1	12.74	0.325
SW 1573	0.2739	1	12.25	0.3144
PK-45H	0.5156	1	14.3	0.3087
22Rv1	0.4516	1	13.55	0.3028
JeKo-1	0.2651	1	13.52	0.2971
JURKAT	0.1047	1	13.08	0.2918
EBC-1	0.7769	1	14.56	0.2899
Panc 02.03	0.2046	1	12.84	0.2888
HCC1937	0.4133	1	14.01	0.287
TUHR10TKB	0.2407	1	13.6	0.2835
HCC-1171	0.1509	1	12.55	0.2764
KU-19-19	0.2386	1	12.66	0.2715
JHH-4	0.1369	1	12.39	0.2656
SW 900	0.1975	1	13.28	0.2641
KYSE-180	0.1718	1	13.76	0.253
Daoy	0.2536	1	13.37	0.2258
SNU-738	0.0539	1	13.23	0.2235
CAL-120	0.5689	1	12.79	0.2158
HCC-1195	0.6807	1	14.05	0.2144
KYSE-150	0.3389	1	12.58	0.2007
RD-ES	0.4079	1	13.56	0.1893
ES-2	0.2956	1	13.02	0.1797
RERF-LC-Ad2	0.3581	1	12.19	0.1752
BCP-1	0.5504	1	12.98	0.174
TM-31	0.5337	1	14.01	0.1725
OVTOKO	0.2482	1	12.02	0.1569
NCI-H1184	0.1936	1	13.39	0.1529
SNU-878	0.2784	1	12.08	0.1505
WM1799	0.1073	1	13.6	0.1458
JHOM-2B	0.5758	1	12.4	0.1427
KYSE-30	0.2614	1	10.41	0.1408
HCC1954	0.4655	1	13.11	0.1365
SK-MEL-31	0.9662	1	13.81	0.1347
NCI-H2106	0.2784	1	13.32	0.1323
SU-DHL-8	0.4596	1	13.52	0.1272
T.T	0.5736	1	12.7	0.127
Hs 746T	0.3112	1	14.02	0.1229
CAL-85-1	0.1446	1	12.3	0.1132
NCI-H3255	0.21	1	13.38	0.1117
HDQ-P1	0.329	1	12.33	0.09524
647-V	0.2485	1	13.3	0.08093
Hep 3B2.1-7	0.4886	1	13.38	0.07612
AMO-1	0.382	1	12.98	0.05719
SW1463	0.245	1	11.01	0.05516
COR-L279	0.2137	1	12.48	0.04521

UM-UC-3	0.5341	1	13.78	0.03979
JHOC-5	0.4874	1	13.01	0.03629
HCC-2279	0.5563	1	13.97	0.03237
OVCAR-8	0.2913	1	13.54	0.03218
WSU-DLCL2	0.074	1	11.36	0.03168
SK-ES-1	0.4537	1	14.27	0.02868
SR-786	0.2108	1	12.98	0.02812
FU97	0.676	1	13.16	0.02744
SW579	0.1552	1	14.26	0.02199
SNU-119	0.4831	1	13.51	0.02139
NCI-H727	0.1042	1	13.32	0.01799
OAW28	0.3236	1	14.13	0.01741
SIG-M5	0.2376	1	12.29	0.0128
SNU-685	0.8653	1	12.62	0.004935
COV644	0.2646	1	11.47	0.003567
59M	0.2938	1	12.54	−0.005838
CAL-29	0.3935	1	13.49	−0.01914
MOLM-16	0.2036	1	13.21	−0.0232
T24	0.053	1	13.92	−0.03142
COV362	0.2828	1	13.67	−0.04908
SK-BR-3	0.0909	1	12.79	−0.05003
COLO-678	0.3672	1	12.71	−0.05769
OE21	0.3278	1	12.5	−0.07898
SNU-503	0.5085	1	12.59	−0.09216
COV318	0.113	1	12.37	−0.09539
A-704	0.5747	1	12.02	−0.1026
BxPC-3	0.2213	1	13.17	−0.1048
PL-21	0.6753	1	13.94	−0.1177
LoVo	0.4156	1	13.75	−0.1183
JHH-1	0.2608	1	11.9	−0.119
NCI-H2073	0.1176	1	13.05	−0.1208
KYSE-270	0.4825	1	12.14	−0.1262
DOHH-2	0.0463	1	12.67	−0.137
NCI-H1648	0.2803	1	13.23	−0.1436
SW480	0.2838	1	12.33	−0.1454
KP-2	0.5908	1	11.66	−0.1583
Malme-3M	0.1366	1	13.16	−0.1588
TEN	0.2695	1	13.24	−0.1602
Detroit 562	0.4213	1	13.54	−0.1705
NU-DHL-1	0.8047	1	12.67	−0.1794
BT-20	0.2627	1	14.53	−0.1984
SU-DHL-4	0.5176	1	13.12	−0.2005
LC-1F	0.1801	1	12.72	−0.2012
SNU-349	0.3539	1	12.6	−0.2043
HT-1197	0.2347	1	11.94	−0.2197
HEL 92.1.7	0.2514	1	12.75	−0.2444
JVM-3	0.6003	1	11.77	−0.2472
ZR-75-1	0.9032	1	11.97	−0.2484
ACHN	0.7081	1	13.35	−0.2549
COLO-680N	0.2382	1	11.81	−0.2591
EFE-184	0.2526	1	11.48	−0.2679
IGR-39	0.5236	1	13.74	−0.2749
RVH-421	0.1231	1	12.4	−0.2787
HMC-1-8	0.2031	1	12.84	−0.2898
A101D	0.1951	1	13.22	−0.3102
JHOM-1	0.4958	1	12.33	−0.3162
OS-RC-2	0.3302	1	12.37	−0.3227
Ishikawa (Heraklio) 02 ER-	0.4566	1	13.5	−0.3289
KP-3	0.5221	1	12.23	−0.3301
Caki-2	0.1023	1	12.26	−0.3463
SNU-1076	0.7478	1	12.31	−0.3531

HCC827	0.4692	1	13.22	−0.3576
SCC-25	0.1755	1	12.76	−0.3592
HSC-2	0.5866	1	12.77	−0.3656
MDA-MB-361	0.5833	1	12.25	−0.3673
OVMANA	0.4446	1	9.114	−0.4041
HSC-4	0.1654	1	12.22	−0.4073
MDA-MB-175-VII	0.2662	1	11.98	−0.408
BHY	0.2464	1	11.31	−0.4115
NCI-H2081	0.3786	1	14.05	−0.4172
NCI-H1339	0.4701	1	13.39	−0.4731
AU565	0.1846	1	13.12	−0.5102
EC-GI-10	0.4115	1	10.63	−0.5198
Panc 08.13	0.5662	1	13.5	−0.5348
GSU	0.1631	1	11.9	−0.5584
HPAC	0.5224	1	11.62	−0.5627
RT4	0.38	1	13.47	−0.569
Calu-1	0.6097	1	14.56	−0.5782
SK-HEP-1	0.4375	1	13.37	−0.5868
NCI-H2170	0.528	1	12.81	−0.5887
TUHR4TKB	0.3347	1	10.4	−0.5985
L-1236	0.4455	1	12.73	−0.6079
PLC/PRF/5	0.2219	1	13.05	−0.6151
NUGC-4	0.3634	1	11.57	−0.6186
BT-483	0.3718	1	11.86	−0.6283
BHT-101	0.205	1	10.31	−0.6403
TE-10	0.1123	1	12.39	−0.6527
KG-1-C	0.157	1	12.3	−0.6657
HSC-3	0.2837	1	13.07	−0.7204
H4	0.383	1	12.86	−0.7459
CL-40	0.5078	1	11.86	−0.7622
RCC10RGB	0.321	1	11.91	−0.7668
A549	0.3624	1	12.84	−0.779
RERF-LC-KJ	0.3155	1	12	−0.84
NCI-H2052	0.136	1	12.53	−0.867
NCI-H2023	0.2448	1	13.08	−0.89
ME-1	0.0866	1	12.08	−0.9049
UACC-62	0.2209	1	11.45	−0.956
NCI-H2110	0.2944	1	12.98	−0.9794
BT-474	0.0951	1	12.46	−0.9845
BICR 6	0.1664	1	11.13	−0.9929
MOLP-2	0.5556	1	10.37	−1.041
MFE-280	0.1962	1	12.6	−1.056
NCI-H322	0.2826	1	13.84	−1.148
OV-90	0.1623	1	12.23	−1.173
NCI-H2444	0.5733	1	13.24	−1.27
JHH-5	0.1391	1	12.64	−1.271
RKO	0.1795	1	12	−1.61
NCI-H1944	0.3785	1	13.48	−1.679
SCaBER	0.2962	1	13.27	−1.774
PA-TU-8902	1.785	2	16.14	1.971
NCI-H1703	0.9498	2	14.66	1.63
IGR-1	1.37	2	14.22	1.567
NCI-H23	0.9978	2	14.03	1.533
HCC-33	1.145	2	14.18	1.346
NCI-H1876	0.7444	2	13.3	1.184
DMS 53	1.066	2	13.68	1.069
SNU-46	0.6093	2	13.82	1.057
MDA-MB-436	0.8725	2	13.79	1.021
FaDu	0.707	2	13.66	0.9121
TE-6	1.073	2	13.02	0.9024
T84	0.6687	2	13.69	0.8607

G-292, clone A141B1	0.6289	2	14.59	0.8524
LS411N	0.5093	2	13.59	0.8354
JHOS-2	1.066	2	13.21	0.8041
HDLM-2	0.9832	2	13.58	0.7641
HCC1428	0.9916	2	14.2	0.6687
697	0.1042	2	13.16	0.6285
ONCO-DG-1	0.5144	2	13.06	0.6274
SK-LU-1	0.8149	2	13.81	0.5177
IA-LM	1.361	2	13.67	0.4917
LS123	0.8414	2	13.03	0.4674
Raji	0.2279	2	13.4	0.4488
Caov-3	0.789	2	13.37	0.4428
NB-1	0.5544	2	14	0.4253
SH-10-TC	1.099	2	13.89	0.3887
TE-5	0.629	2	12.63	0.3498
LCLC-97TM1	0.4523	2	12.72	0.2582
MUTZ-5	0.1093	2	12.98	0.2129
LS1034	1.197	2	13.07	0.2022
SJSA-1	0.2343	2	13.2	0.1093
COR-L105	0.7669	2	13.58	0.1025
BICR 16	0.6912	2	13.14	0.03338
FU-OV-1	0.681	2	13.29	−0.03554
Reh	0.0981	2	12.79	−0.1594
NCI-H226	0.5821	2	13.36	−0.292
KP4	0.9934	2	13.62	−0.3814
Hey-A8	0.5776	2	12.24	−0.4531
NCI-H1573	0.849	2	13.37	−0.5031
SU.86.86	0.9101	2	11.53	−0.5191
SK-MEL-1	1.321	2	14.84	−0.5814

Table S3. RNA-seq differential expression analysis related to Figure 9C–F. Lists of differentiallyexpressed genes for FOXM1 isoforms in HEK293T FOXM1 KO cells.

Gene	log2(Fold Change)	*p* Value	FDR
HIST1H2BC	1.65917	5.00E−05	0.0129821
C17orf91, MIR22	1.32201	5.00E−05	0.0129821
LOC100129203	1.21075	0.00015	0.0302917
LOC648987	0.782979	5.00E−05	0.0129821
NEURL1B	0.771869	5.00E−05	0.0129821
RN7SK	0.750412	0.0001	0.0234516
LMO7	0.671486	5.00E−05	0.0129821
INPP5D	0.664307	0.0002	0.0354634
GJA3	0.63615	5.00E−05	0.0129821
MAFF	0.618286	0.00015	0.0302917
CDKN3	0.57	5.00E−05	0.0129821
ZNF823	0.561693	0.0002	0.0354634
PRPH	0.542506	5.00E−05	0.0129821
CASP10	0.54155	0.00025	0.0417816
STEAP2	0.534012	5.00E−05	0.0129821
TROAP	0.516808	5.00E−05	0.0129821
CDC25B	0.512772	5.00E−05	0.0129821
PRRT3	0.510336	5.00E−05	0.0129821
GAS1	0.508725	5.00E−05	0.0129821
BCYRN1	0.502052	0.0002	0.0354634
DSC3	0.498324	5.00E−05	0.0129821
ARL6IP1	0.491064	5.00E−05	0.0129821
C10orf140	0.482816	5.00E−05	0.0129821
MICB	0.48175	5.00E−05	0.0129821
HP1BP3	0.473816	5.00E−05	0.0129821
HERC5	0.460507	5.00E−05	0.0129821

GAS2L3	0.455805	5.00E−05	0.0129821
PCDH10	0.450132	5.00E−05	0.0129821
CPSF1, MIR939	0.430302	0.00025	0.0417816
PIK3CA	0.425154	5.00E−05	0.0129821
FAM100B	0.418678	5.00E−05	0.0129821
PTTG1	0.412861	5.00E−05	0.0129821
LOC440926	0.3959	0.0001	0.0234516
TNFAIP8L1	0.395495	5.00E−05	0.0129821
CCNB1	0.393968	5.00E−05	0.0129821
TMEM231	0.388	0.00015	0.0302917
ZNF514	0.383139	0.0001	0.0234516
FLJ39632	0.37633	5.00E−05	0.0129821
SLC43A2	0.372264	5.00E−05	0.0129821
PAIP2B	0.363829	5.00E−05	0.0129821
CRLF3	0.35399	5.00E−05	0.0129821
KIAA1161	0.347517	0.0001	0.0234516
GTSE1	0.342078	0.00015	0.0302917
ARHGAP21	0.325421	0.0002	0.0354634
TRIM59	0.324268	5.00E−05	0.0129821
FOXG1	0.316912	5.00E−05	0.0129821
CDK2AP2	0.311131	0.0001	0.0234516
ZBTB7A	0.302069	0.00015	0.0302917
LOC440157	0.300733	0.0002	0.0354634
NDNF	0.292996	0.0002	0.0354634
DCLRE1C	0.289365	0.00015	0.0302917
LOC100506453	0.287278	0.00015	0.0302917
EGLN3	0.273048	5.00E−05	0.0129821
PIM1	0.269906	0.00015	0.0302917
CENPA	0.254407	0.00025	0.0417816
SCAPER	−0.263648	0.0003	0.0490112
COBLL1	−0.301084	0.0003	0.0490112
TMEM39B	−0.307412	0.00025	0.0417816
CCDC130	−0.313858	0.0002	0.0354634
CPEB4	−0.316257	0.00015	0.0302917
POC5	−0.332147	5.00E−05	0.0129821
C16orf87	−0.334683	5.00E−05	0.0129821
CAV2	−0.338002	5.00E−05	0.0129821
THUMPD2	−0.338846	5.00E−05	0.0129821
LOC388796	−0.342756	5.00E−05	0.0129821
GADD45A	−0.353708	5.00E−05	0.0129821
AEN	−0.357223	0.00015	0.0302917
RPGRIP1L	−0.360572	5.00E−05	0.0129821
ZRSR2	−0.362461	0.0002	0.0354634
NFKBIL1	−0.368066	5.00E−05	0.0129821
ZNF510	−0.393861	0.0002	0.0354634
RASSF1	−0.39517	5.00E−05	0.0129821
KRCC1	−0.415031	0.0001	0.0234516
C7orf40	−0.456667	5.00E−05	0.0129821
TMEM198	−0.477247	0.00025	0.0417816
KRTAP19−1	−0.482781	5.00E−05	0.0129821
CCT6P3	−0.549323	5.00E−05	0.0129821
PHF21B	−0.646559	5.00E−05	0.0129821
DUSP8	−0.652271	5.00E−05	0.0129821
WDR78	−0.770678	0.0002	0.0354634
RN7SL1	−0.789177	5.00E−05	0.0129821
PVT1	−0.839657	5.00E−05	0.0129821
OVGP1	−0.892928	5.00E−05	0.0129821
RN7SL2	−0.94686	5.00E−05	0.0129821
TCEAL7	−0.996853	5.00E−05	0.0129821
C10orf118, MIR2110	−2.34444	5.00E−05	0.0129821
GH1	−2.9968	5.00E−05	0.0129821

Table S4. Lists of genes in Venn Diagram related to Figure 9E. Lists of genes showing both increased and decreased expression and overlap for FOXM1 isoforms in HEK293T FOXM1 KO cells.

**FOXM1a FOXM1b FOXM1c**	**FOXM1a FOXM1b**	**FOXM1a FOXM1c**	**FOXM1b FOXM1c**	**FOXM1a**	**FOXM1b**	**FOXM1c**
ZNF823	MIR564, TMEM42	DUSP8	KRCC1	IFIT1	GRIN2D	TMEM198
PHF21B	RSBN1		THUMPD2	LATS1	FAM172A, MIR2277	COBLL1
RN7SL1	MIR4723, TMEM199		GAS1	WDR96	ZNF295	CENPA
OVGP1			KRTAP19-1	CCL5	PPARGC1A	LOC388796
C10orf118, MIR2110			CPSF1, MIR939	HMOX1	ACYP1	TNFAIP8L1
RN7SL2			RN7SK	HPS1, MIR4685	TNFSF9	HERC5
EGLN3			AEN	SCAND3	MIR4749, PTOV1	LOC100129203
GH1			ZNF514	PLSCR1	C8orf83	TRIM59
NEURL1B			LOC100506453	IFITM1	CSPG4	MICB
			LMO7	IRF9	IGFBP4	CCNB1
			KIAA1161	HSPA1B	TRIT1	C16orf87
			SCAPER	LGALS3BP	DBP	CDK2AP2
			PVT1	DDX60	SOX9	GAS2L3
			DSC3	PRIC285	LENG8	CPEB4
			HIST1H2BC	FGD6	MIR943, WHSC2	TMEM39B
			CCT6P3	DDX58	SPRED2	BCYRN1
			ARL6IP1	LOC285074	KLF10	C17orf91, MIR22
			PCDH10	MIR1909, REXO1	SPRYD7	GTSE1
			CDKN3	IFI6	TEX14	CCDC130
			CDC25B	ARNTL	NBEAL2	ARHGAP21
			SLC43A2	CTC1	BCAM	ZNF510
			NFKBIL1	DGKH	DNAL4	ZRSR2
			POC5	INHBE	PITPNM2	C10orf140
			HP1BP3	FAM154B	G2E3	MAFF
			C7orf40	ZNF425	FAT4	STEAP2
			TROAP	ISG15	IGF1R	CRLF3
			FAM100B	IFIT3	TRIM45	PRRT3
			RPGRIP1L	GALK2	ADCY6, MIR4701	GADD45A
			GJA3	LOC100288637	CENPE	TMEM231
			FLJ39632	PLAC2	MFSD9	RASSF1
			LOC440157	ARID4A	LOC100507460	PTTG1
			DCLRE1C	SAMD9	PCNXL2	TCEAL7
			PRPH		PI15	CAV2
			PIK3CA		MN1	NDNF
			FOXG1		EVC	CASP10
			PIM1		PIK3C2B	LOC440926
			INPP5D		FLJ14186	ZBTB7A
					FBXL20	PAIP2B
					SUPT3H	LOC648987
					SLC38A7	WDR78
					MIR3615, SLC9A3R1	
					KRT8	
					KIAA0226, MIR922	
					CEP89	

CELSR3, MIR4793
C1orf152
ZNF79
CCNF
COL1A1
MESDC1
GULP1
ENO2
KIF20A
SPDYE7P
TM2D1
ANKRD54, MIR658
EGFR
PABPC1L
ZNF148
CCDC76
SETD1B
TP53I3
TMEM100
ZNF343
RAPH1
UCP2
FBLN5
WDR74
NCRNA00287
DEPDC1
DHRS2
FLJ45340

Table S5. Lists of genes in Venn Diagram related to Figure 9F. Lists of genes showing increased expression and overlap for FOXM1 isoforms in HEK293T FOXM1 KO cells.

FOXM1a FOXM1b FOXM1c	FOXM1a FOXM1b	FOXM1b FOXM1c	FOXM1a	FOXM1b	FOXM1c
ZNF823	MIR472, TMEM199	CDKN3	DDX60	FBXL20	ARHGAP21
EGLN3		GAS1	PRIC285	GRIN2D	CENPA
NEURL1B		CDC25B	DDX58	SLC38A7	CPSF1, MIR939
		RN7SK	IFIT1	MIR564, TMEM42	TNFAIP8L1
		SLC43A2	IFI6	MIR3615, SLC9A3R1	C10orf140
		ZNF514	CCL5	KRT8	MAFF
		LOC100506453	CTC1	C1orf152	STEAP2
		LMO7	HMOX1	TNFSF9	HERC5
		HP1BP3	HPS1, MIR4685	MIR4749, PTOV1	CRLF3
		KIAA1161	PLSCR1	CSPG4	PRRT3
		TROAP	IFITM1	IGFBP4	TMEM231
		FAM100B	INHBE	CCNF	LOC100129203
		GJA3	ISG15	DBP	PTTG1
		FLJ39632	IRF9	SOX9	TRIM59
		DSC3	IFIT3	LENG8	MICB
		LOC440157	HSPA1B	MIR943, WHSC2	CCNB1
		DCLRE1C	LGALS3BP	COL1A1	CDK2AP2
		HIST1H2BC	SAMD9	SPRED2	GAS2L3
		PRPH		MESDC1	NDNF

PIK3CA	ENO2	BCYRN1
FOXG1	TEX14	C17orf91, MIR22
PIM1	NBEAL2	CASP10
ARL6IP1	BCAM	LOC440926
INPP5D	KIF20A	GTSE1
PCDH10	SPDYE7P	ZBTB7A
	PITPNM2	PAIP2B
	FAT4	LOC648987
	EGFR	
	PABPC1L	
	IGF1R	
	TRIM45	
	ADCY6, MIR4701	
	SETD1B	
	TP53I3	
	MFSD9	
	LOC100507460	
	PCNXL2	
	RAPH1	
	UCP2	
	FBLN5	
	MN1	
	EVC	
	PIK3C2B	
	NCRNA00287	
	WDR74	
	DHRS2	
	FLJ14186	
	FLJ45340	

Table S6. Lists of primers and sequences.

**Target**	**Application**	**Forward Sequence**
Pan-FOXM1 F	RT-PCR, RT-qPCR	GCAGGCTGCACTATCAACAA
Pan-FOXM1 R	RT-PCR, RT-qPCR	TCGAAGGCTCCTCAACCTTA
FOXM1a F	RT-qPCR	TGGGGAACAGGTGGTGTTTGG
FOXM1a R	RT-qPCR	GCTAGCAGCACTGATAAACAAAG
FOXM1b F	RT-qPCR	CCAGGTGTTTAAGCAGCAGA
FOXM1b R	RT-qPCR	TCCTCAGCTAGCAGCACCTTG
FOXM1c F	RT-qPCR	CAATTGCCCGAGCACTTGGAATCA
FOXM1c R	RT-qPCR	TCCTCAGCTAGCAGCACCTTG
FOXM1 UTR F	RT-qPCR	TCCCTGCTGCCTGATTATGC
FOXM1 UTR R	RT-qPCR	TCACCATTGCCTTTGTTGTTCC
FOXM1 EX1/2 F	RT-qPCR	GTCTGGAGGGTCCACACTTG
FOXM1 EX1/2 R	RT-qPCR	CTTGGAGGCCTCTGCTTGAT
CCNB1 F	RT-qPCR	AACTTTCGCCTGAGCCTATTTT
CCNB1 R	RT-qPCR	TTGGTCTGACTGCTTGCTCTT
CCNE1 F	RT-qPCR	GAAATGGCCAAAATCGACAG
CCNE1 R	RT-qPCR	TCTTTGTCAGGTGTGGGGA
18s rRNA F	RT-PCR, RT-qPCR	CAGCCACCCGAGATTGAGCA
18s rRNA R	RT-PCR, RT-qPCR	TAGTAGCGACGGGCGGTGTG
FOXM1 KO F	PCR	ATCTCCAACCCAGTCCCATTTT
FOXM1 KO R	PCR	CCTGGCTTGGGGACGTCTAT
FOXM1 WT F	PCR	GCAAGAGTTGGACTCTGCCA
FOXM1 WT R	PCR	ATCTCCAACCCAGTCCCATTTT
FOXM1_QC1 F	Site-directed mutagenesis	CCATCCGCCACAACCTTGCCCTGCACGAC
FOXM1_QC1 R	Site-directed mutagenesis	GTCGTGCAGGGCAAGGTTGTGGCGGATGG
FOXM1_QC2 F	Site-directed mutagenesis	GCTGGAAGAACTCCATCGCCGCCAACCTTGCCCTGCACG
FOXM1_QC2 R	Site-directed mutagenesis	CGTGCAGGGCAAGGTTGGCGGCGATGGAGTTCTTCCAGC

© 2019 by the authors. Licensee MDPI, Basel, Switzerland. This article is an open access article distributed under the terms and conditions of the Creative Commons Attribution (CC BY) license (http://creativecommons.org/licenses/by/4.0/).
